# Correction: Splenocytes Seed Bone Marrow of Myeloablated Mice: Implication for Atherosclerosis

**DOI:** 10.1371/journal.pone.0137264

**Published:** 2015-09-01

**Authors:** The PLOS ONE Staff

## Notice of Republication

This article was republished on August 14, 2015, to correct errors in the order of the figures. The publisher apologizes for the errors. Please download this article again to view the correct version. The originally published, uncorrected article and the republished, corrected article are provided here for reference.

## Supporting Information

S1 File Originally published, uncorrected article. (PDF)

S2 File Republished corrected article. (PDF)
