# Supplementary material for: Splenocytes Seed Bone Marrow of Myeloablated Mice: Implication for Atherosclerosis
Source: PLoS One. 2015 Jun 3;10(6):e0125961. doi: 10.1371/journal.pone.0125961 (PMC4454495; doi:10.1371/journal.pone.0125961)
Supplement: S1 Fig — DNA was extracted from the indicated recipient mouse tissues, amplified by PCR followed by analysis of PCR products by 1.5% agarose gel. The upper bands (395 bp) correspond to the amplified Y chromosome segment and the lower bands (401 bp) is the internal control (β globin). The male (M) and female (F) brain cDNAs were used as positive and negative controls for Y chromosome. Distilled water was used for PCR control. (PPTX) [file pone.0125961.s001.pptx]

## Slide 1
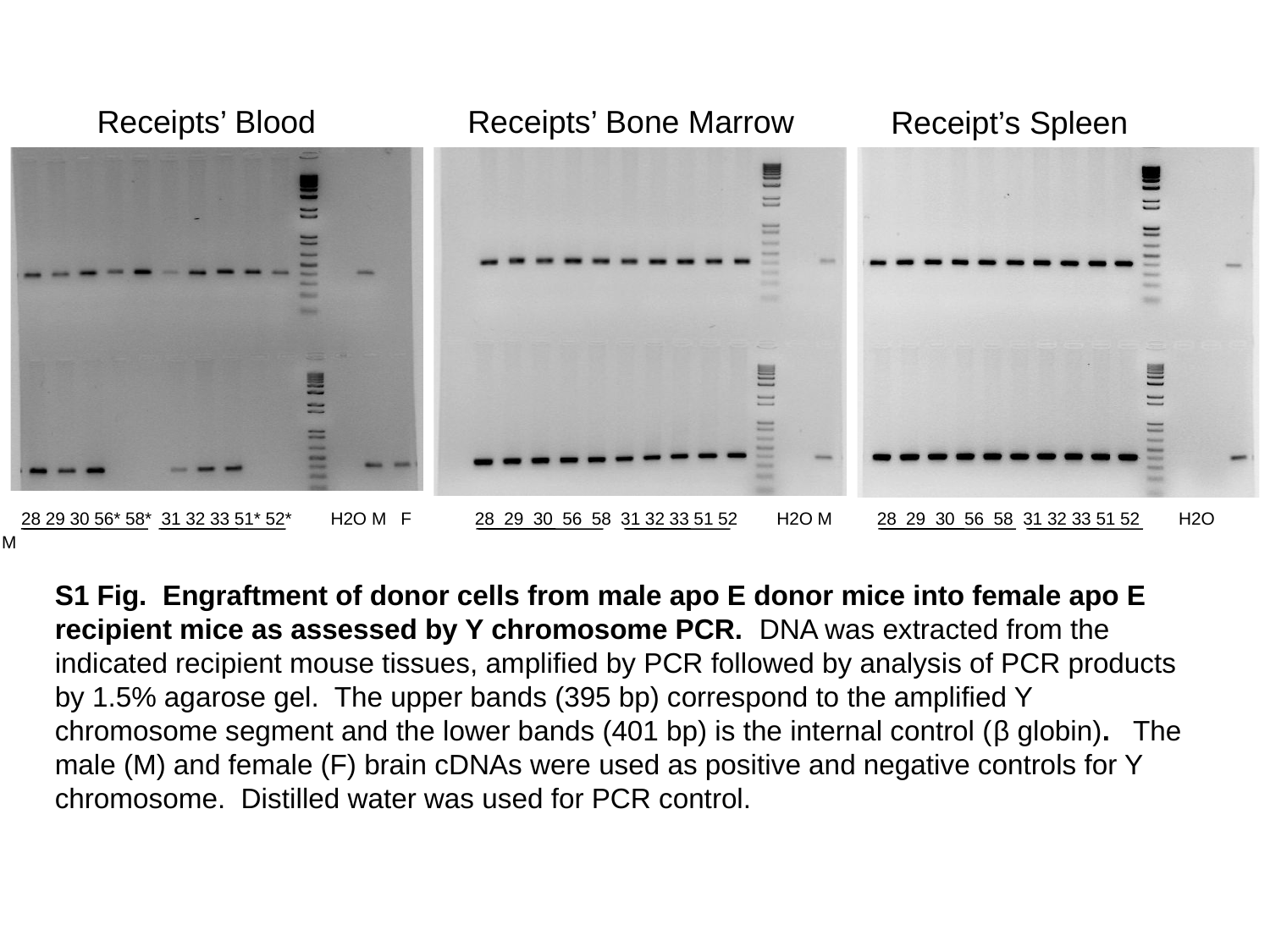

Receipts’ Blood
Receipts’ Bone Marrow
Receipt’s Spleen
 28 29 30 56* 58* 31 32 33 51* 52* H2O M F 28 29 30 56 58 31 32 33 51 52 H2O M 28 29 30 56 58 31 32 33 51 52 H2O M
S1 Fig. Engraftment of donor cells from male apo E donor mice into female apo E recipient mice as assessed by Y chromosome PCR. DNA was extracted from the indicated recipient mouse tissues, amplified by PCR followed by analysis of PCR products by 1.5% agarose gel. The upper bands (395 bp) correspond to the amplified Y chromosome segment and the lower bands (401 bp) is the internal control (β globin). The male (M) and female (F) brain cDNAs were used as positive and negative controls for Y chromosome. Distilled water was used for PCR control.
