# Supplementary material for: Splenocytes Seed Bone Marrow of Myeloablated Mice: Implication for Atherosclerosis
Source: PLoS One. 2015 Jun 3;10(6):e0125961. doi: 10.1371/journal.pone.0125961 (PMC4454495; doi:10.1371/journal.pone.0125961)
Supplement: S2 Fig — Splenocytes and bone marrow were isolated from male donor mice and stained with the indicated antibodies for flow cytometry analysis. Panel A shows the flow cytometry strategy. Panels B, C, and D show analysis of harvested cells for the level of macrophages, eosinophils, and neutrophils, respectively. These data show that the level of macrophages, eosinophils and neutrophils are significantly higher in the bone marrow than in the spleen of C57BL/6 donor mouse. (PPTX) [file pone.0125961.s002.pptx]

## Slide 1
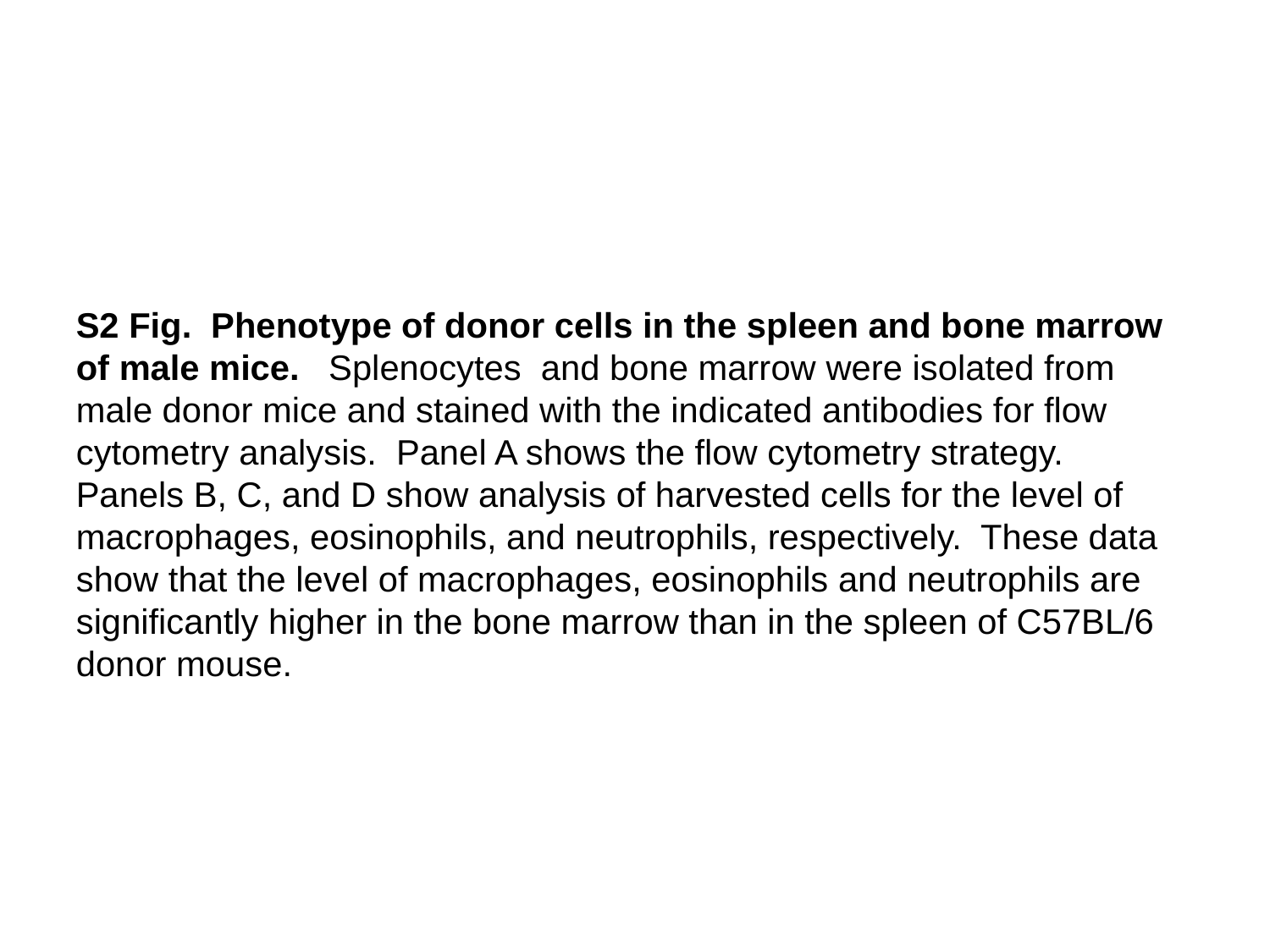

S2 Fig. Phenotype of donor cells in the spleen and bone marrow of male mice. Splenocytes and bone marrow were isolated from male donor mice and stained with the indicated antibodies for flow cytometry analysis. Panel A shows the flow cytometry strategy. Panels B, C, and D show analysis of harvested cells for the level of macrophages, eosinophils, and neutrophils, respectively. These data show that the level of macrophages, eosinophils and neutrophils are significantly higher in the bone marrow than in the spleen of C57BL/6 donor mouse.

## Slide 2
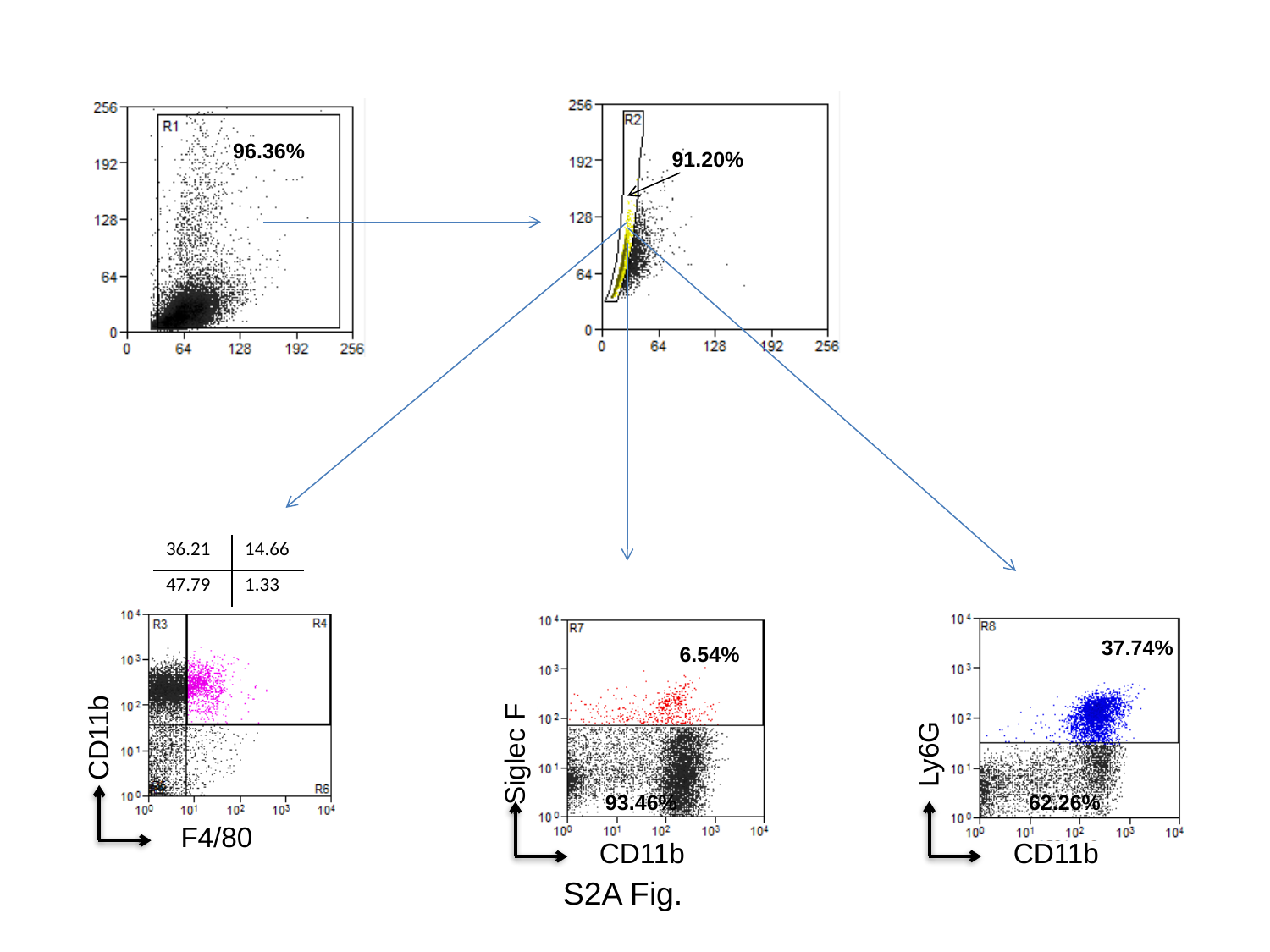

96.36%
91.20%
| 36.21 | 14.66 |
| --- | --- |
| 47.79 | 1.33 |
37.74%
6.54%
CD11b
F4/80
Siglec F
CD11b
Ly6G
CD11b
93.46%
62.26%
S2A Fig.

## Slide 3
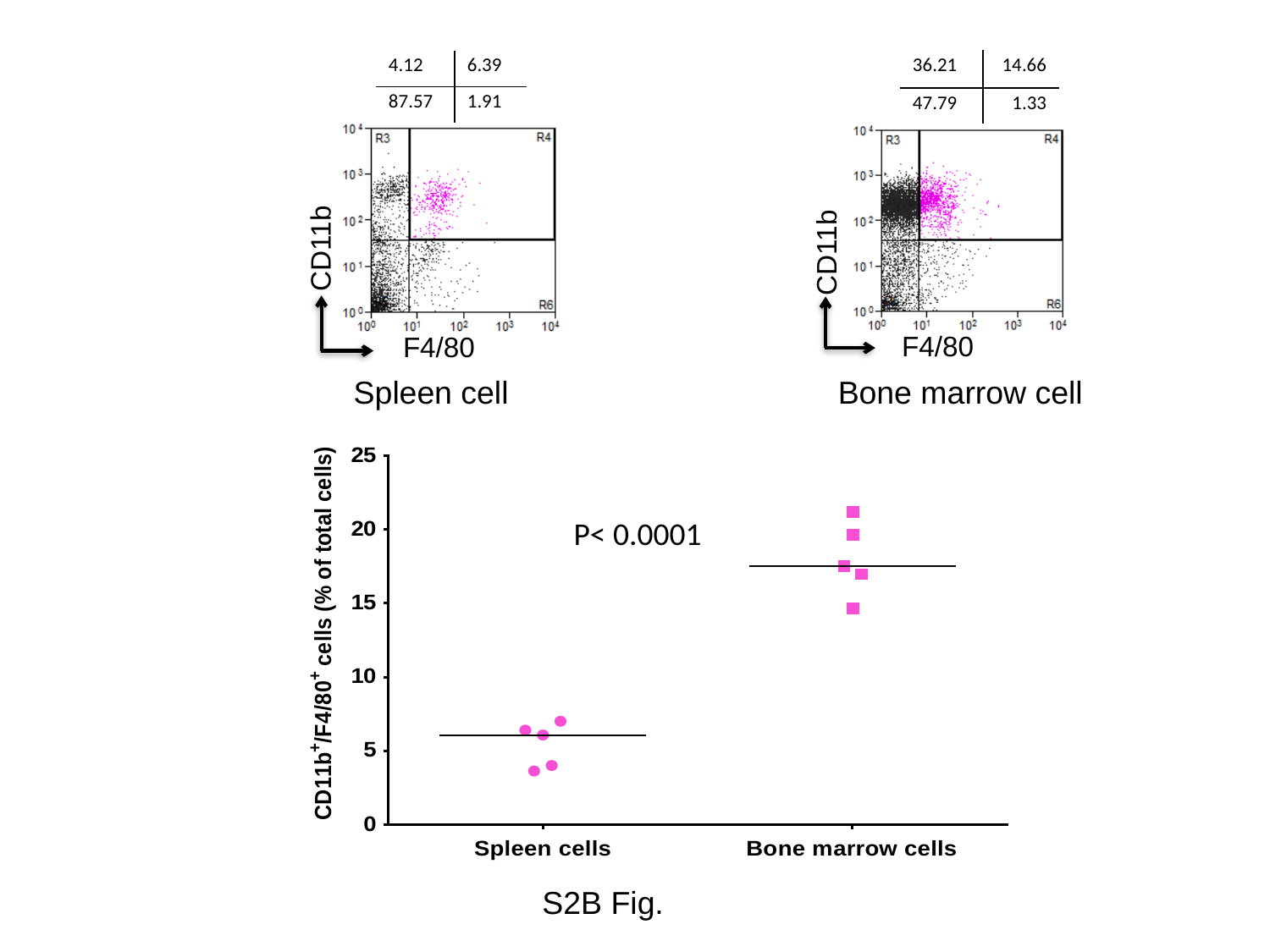

| 36.21 | 14.66 |
| --- | --- |
| 47.79 | 1.33 |
| 4.12 | 6.39 |
| --- | --- |
| 87.57 | 1.91 |
CD11b
F4/80
CD11b
F4/80
Spleen cell
Bone marrow cell
P< 0.0001
S2B Fig.

## Slide 4
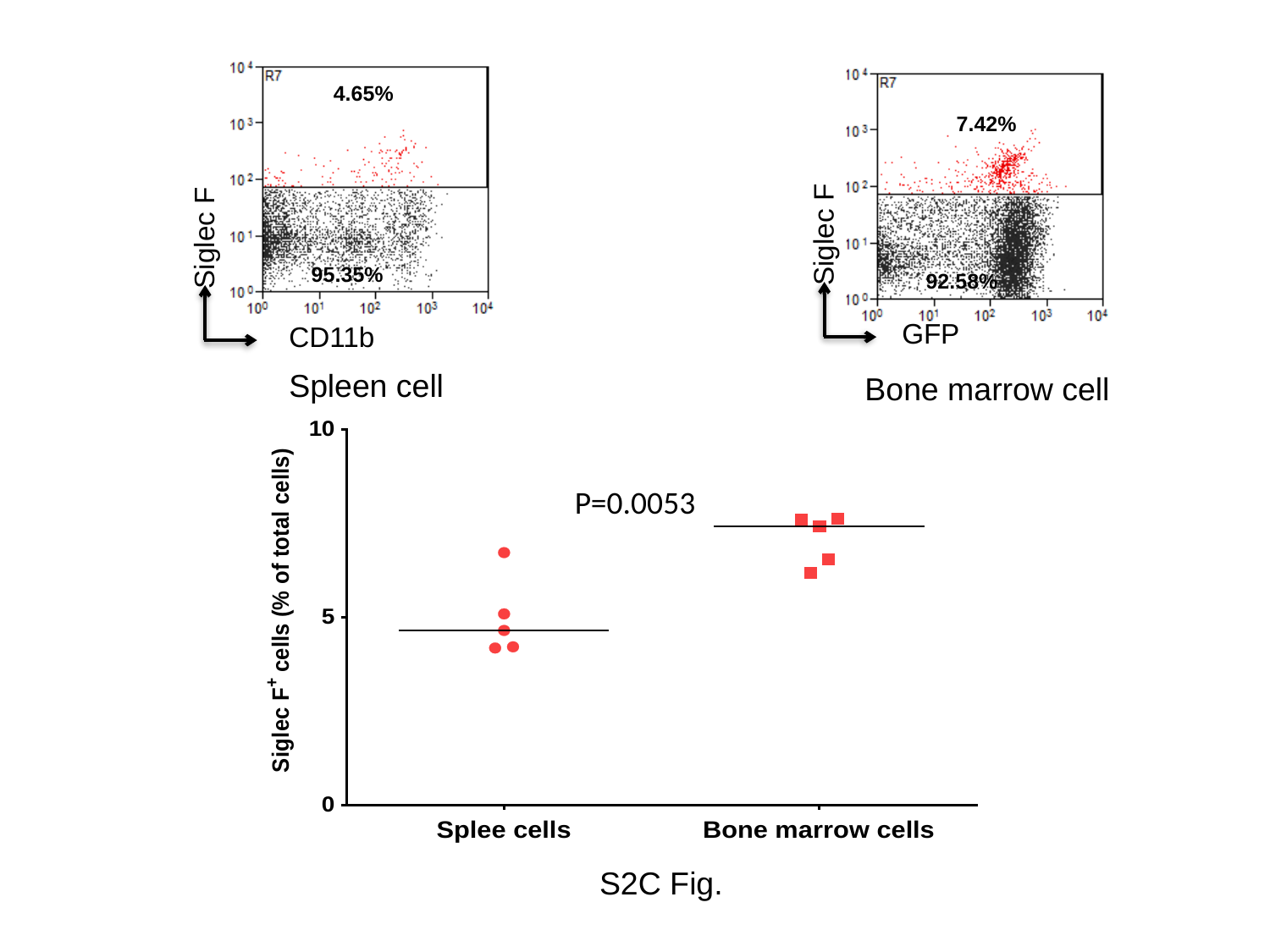

4.65%
7.42%
Siglec F
GFP
Siglec F
CD11b
95.35%
92.58%
Spleen cell
Bone marrow cell
P=0.0053
S2C Fig.

## Slide 5
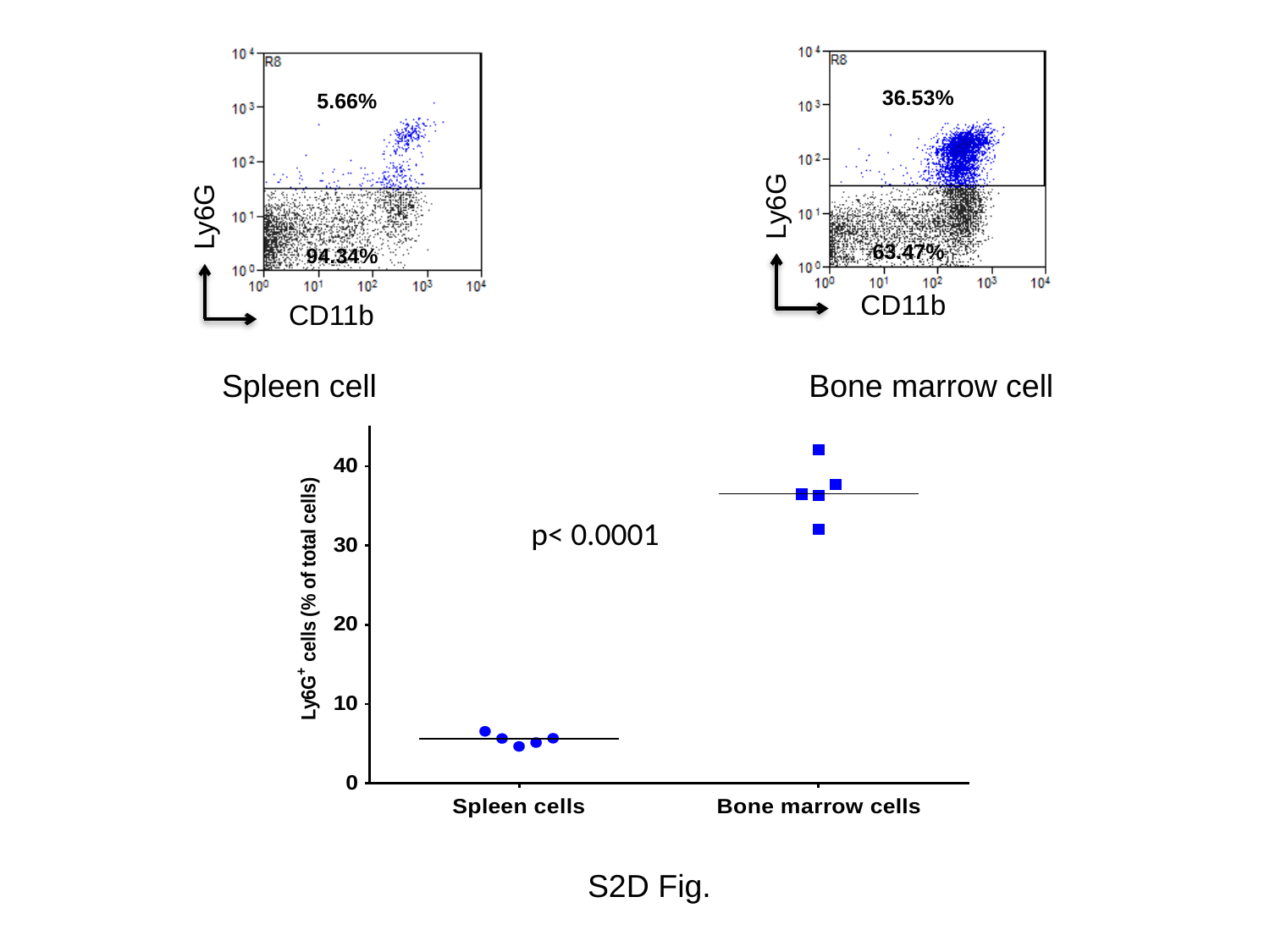

36.53%
5.66%
Ly6G
CD11b
Ly6G
CD11b
63.47%
94.34%
Spleen cell
Bone marrow cell
p< 0.0001
S2D Fig.
