# Supplementary material for: Splenocytes Seed Bone Marrow of Myeloablated Mice: Implication for Atherosclerosis
Source: PLoS One. 2015 Jun 3;10(6):e0125961. doi: 10.1371/journal.pone.0125961 (PMC4454495; doi:10.1371/journal.pone.0125961)
Supplement: S1 Table — (PPTX) [file pone.0125961.s003.pptx]

## Slide 1
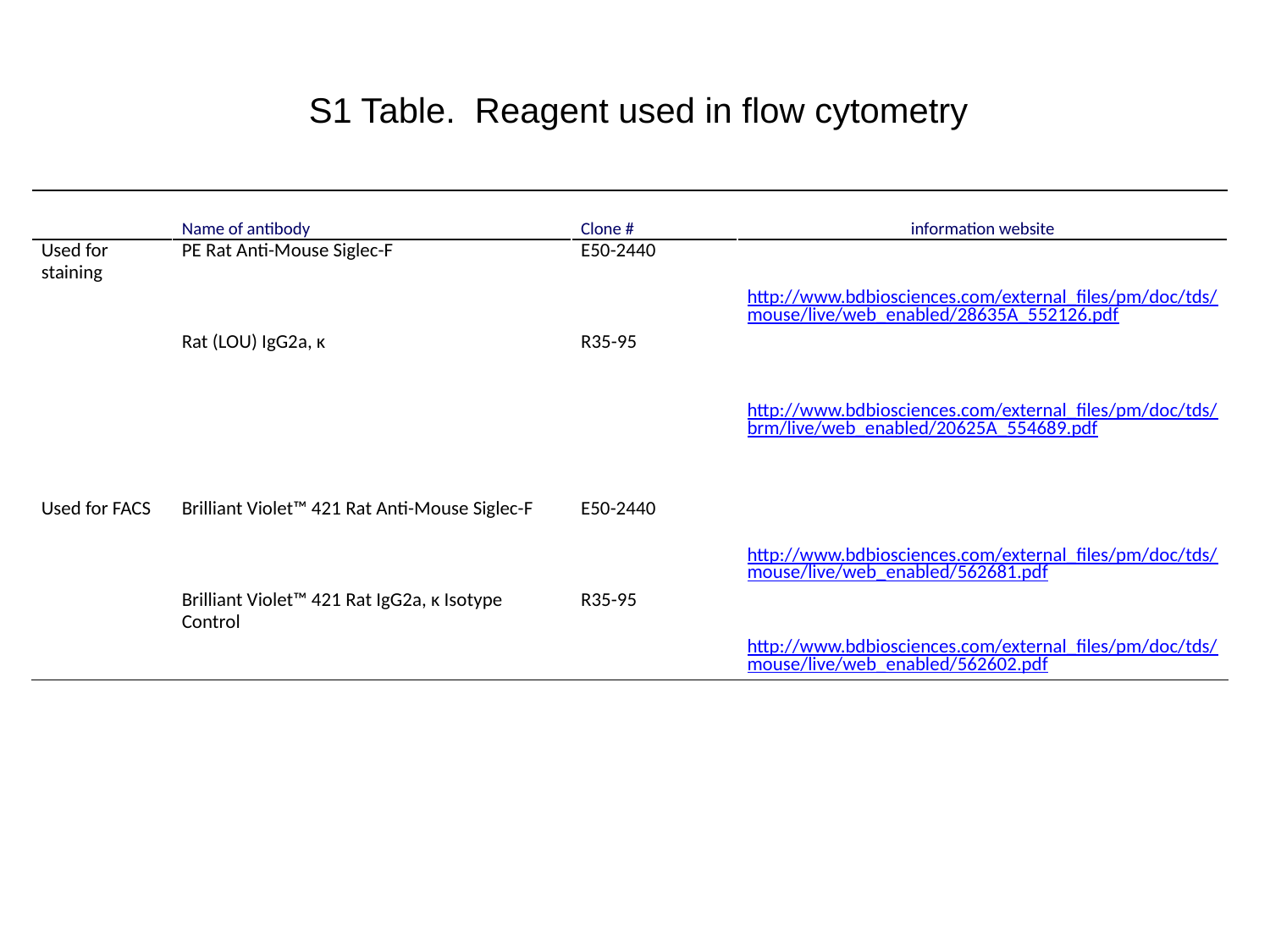

S1 Table. Reagent used in flow cytometry
| | Name of antibody | Clone # | information website |
| --- | --- | --- | --- |
| Used for staining | PE Rat Anti-Mouse Siglec-F | E50-2440 | http://www.bdbiosciences.com/external\_files/pm/doc/tds/mouse/live/web\_enabled/28635A\_552126.pdf |
| | Rat (LOU) IgG2a, κ | R35-95 | http://www.bdbiosciences.com/external\_files/pm/doc/tds/brm/live/web\_enabled/20625A\_554689.pdf |
| | | | |
| Used for FACS | Brilliant Violet™ 421 Rat Anti-Mouse Siglec-F | E50-2440 | http://www.bdbiosciences.com/external\_files/pm/doc/tds/mouse/live/web\_enabled/562681.pdf |
| | Brilliant Violet™ 421 Rat IgG2a, κ Isotype Control | R35-95 | http://www.bdbiosciences.com/external\_files/pm/doc/tds/mouse/live/web\_enabled/562602.pdf |
