# Supplementary material for: Splenocytes Seed Bone Marrow of Myeloablated Mice: Implication for Atherosclerosis
Source: PLoS One. 2015 Jun 3;10(6):e0125961. doi: 10.1371/journal.pone.0125961 (PMC4454495; doi:10.1371/journal.pone.0125961)
Supplement: S2 Table — (PPTX) [file pone.0125961.s004.pptx]

## Slide 1
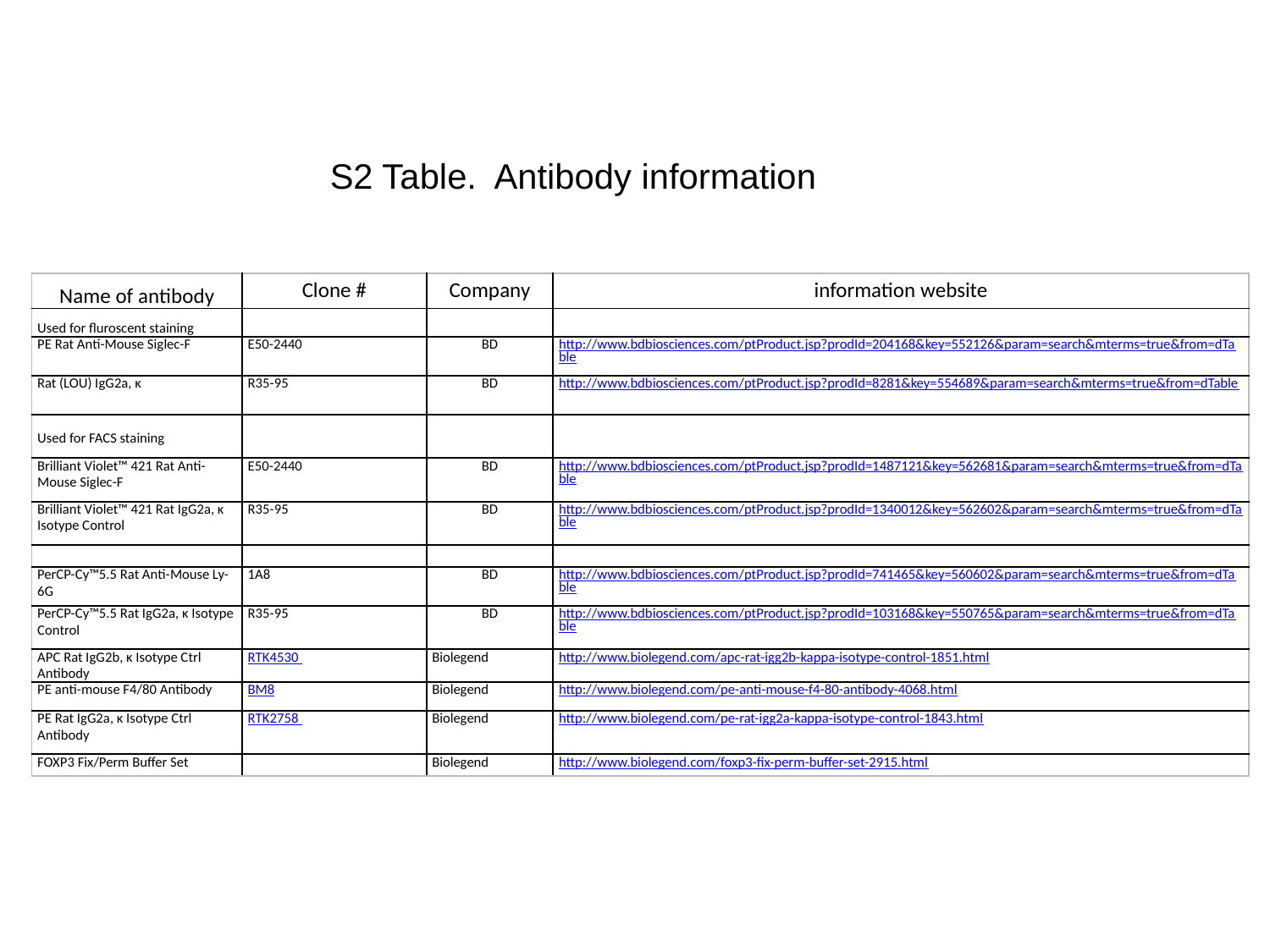

S2 Table. Antibody information
| Name of antibody | Clone # | Company | information website |
| --- | --- | --- | --- |
| Used for fluroscent staining | | | |
| PE Rat Anti-Mouse Siglec-F | E50-2440 | BD | http://www.bdbiosciences.com/ptProduct.jsp?prodId=204168&key=552126&param=search&mterms=true&from=dTable |
| Rat (LOU) IgG2a, κ | R35-95 | BD | http://www.bdbiosciences.com/ptProduct.jsp?prodId=8281&key=554689&param=search&mterms=true&from=dTable |
| Used for FACS staining | | | |
| Brilliant Violet™ 421 Rat Anti-Mouse Siglec-F | E50-2440 | BD | http://www.bdbiosciences.com/ptProduct.jsp?prodId=1487121&key=562681&param=search&mterms=true&from=dTable |
| Brilliant Violet™ 421 Rat IgG2a, κ Isotype Control | R35-95 | BD | http://www.bdbiosciences.com/ptProduct.jsp?prodId=1340012&key=562602&param=search&mterms=true&from=dTable |
| | | | |
| PerCP-Cy™5.5 Rat Anti-Mouse Ly-6G | 1A8 | BD | http://www.bdbiosciences.com/ptProduct.jsp?prodId=741465&key=560602&param=search&mterms=true&from=dTable |
| PerCP-Cy™5.5 Rat IgG2a, κ Isotype Control | R35-95 | BD | http://www.bdbiosciences.com/ptProduct.jsp?prodId=103168&key=550765&param=search&mterms=true&from=dTable |
| APC Rat IgG2b, κ Isotype Ctrl Antibody | RTK4530 | Biolegend | http://www.biolegend.com/apc-rat-igg2b-kappa-isotype-control-1851.html |
| PE anti-mouse F4/80 Antibody | BM8 | Biolegend | http://www.biolegend.com/pe-anti-mouse-f4-80-antibody-4068.html |
| PE Rat IgG2a, κ Isotype Ctrl Antibody | RTK2758 | Biolegend | http://www.biolegend.com/pe-rat-igg2a-kappa-isotype-control-1843.html |
| FOXP3 Fix/Perm Buffer Set | | Biolegend | http://www.biolegend.com/foxp3-fix-perm-buffer-set-2915.html |
