# Supplementary figures and images for: Splenocytes Seed Bone Marrow of Myeloablated Mice: Implication for Atherosclerosis
Source: PLoS One. 2015 Jun 3;10(6):e0125961. doi: 10.1371/journal.pone.0125961 (PMC4454495; doi:10.1371/journal.pone.0125961)

## Slide 1
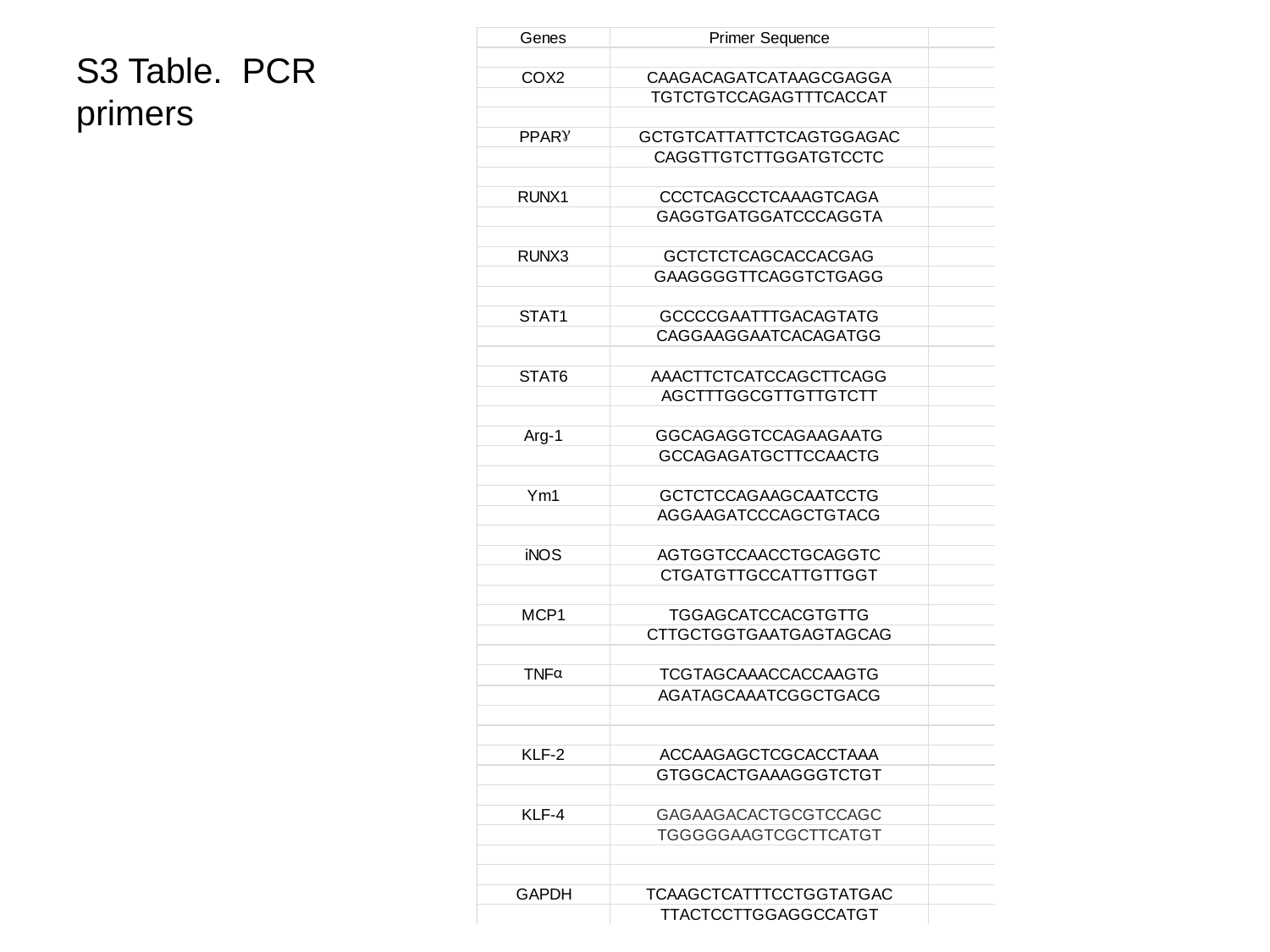

S3 Table. PCR primers

Supplement: S3 Table — (PPTX) [file pone.0125961.s005.pptx]
