# Supplementary material for: Correction: Splenocytes Seed Bone Marrow of Myeloablated Mice: Implication for Atherosclerosis
Source: PLoS One. 2015 Sep 1;10(9):e0137264. doi: 10.1371/journal.pone.0137264 (PMC4556566; doi:10.1371/journal.pone.0137264)
Supplement: S2 File — (PDF) [file pone.0137264.s002.pdf]

RESEARCH ARTICLE

Splenocytes Seed Bone Marrow of Myeloablated Mice: Implication for Atherosclerosis

Lai Wang, Mingjie Yang, Ana Arias, Lei Song, Fuqiang Li, Fang Tian, Minghui Qin, Ada Yukht, Ian K. Williamson, Prediman K. Shah, Behrooz G. Sharifi*

Oppenheimer Atherosclerosis Research Center, Division of Cardiology, Cedars-Sinai Heart Institute, Los Angeles, California, United States of America

* Sharifi@cshs.org

CrossMark
click for updates

OPEN ACCESS

Citation: Wang L, Yang M, Arias A, Song L, Li F, Tian F, et al. (2015) Splenocytes Seed Bone Marrow of Myeloablated Mice: Implication for Atherosclerosis. PLoS ONE 10(6): e0125961. doi:10.1371/journal.pone.0125961

Academic Editor: Christian Schulz, Medical Faculty, Ludwig Maximilians University Munich, GERMANY

Received: December 16, 2014

Accepted: March 28, 2015

Published: June 3, 2015

Copyright: © 2015 Wang et al. This is an open access article distributed under the terms of the Creative Commons Attribution License, which permits unrestricted use, distribution, and reproduction in any medium, provided the original author and source are credited.

Data Availability Statement: All relevant data are within the paper and its Supporting Information files.

Funding: The authors gratefully acknowledge the support of the Heart Foundation, the Eisner Foundation, the Spielberg Foundation, Corday Foundation, and the Skirball Foundation. The evolution of this approach was initially supported by a program project grant from NHLBI grant R01 HL104068.

Competing Interests: The authors have declared that no competing interests exist.

Abstract

Extramedullary hematopoiesis has been shown to contribute to the pathogenesis of a variety of diseases including cardiovascular diseases. In this process, the spleen is seeded with mobilized bone marrow cells that augment its hematopoietic ability. It is unclear whether these immigrant cells that are produced/reprogrammed in spleen are similar or different from those found in the bone marrow. To begin to understand this, we investigated the relative potency of adult splenocytes per se to repopulate bone marrow of lethally-irradiated mice and its functional consequences in atherosclerosis. The splenocytes were harvested from GFP donor mice and transplanted into myeloablated wild type recipient mice without the inclusion of any bone marrow helper cells. We found that adult splenocytes repopulated bone marrow of myeloablated mice and the transplanted cells differentiated into a full repertoire of myeloid cell lineages. The level of monocytes/macrophages in the bone marrow of recipient mice was dependent on the cell origin, i.e., the donor splenocytes gave rise to significantly more monocytes/macrophages than the donor bone marrow cells. This occurred despite a significantly lower number of hematopoietic stem cells being present in the donor splenocytes when compared with donor bone marrow cells. Atherosclerosis studies revealed that donor splenocytes displayed a similar level of atherogenic and atheroprotective activities to those of donor bone marrow cells. Cell culture studies showed that the phenotype of macrophages derived from spleen is different from those of bone marrow. Together, these results demonstrate that splenocytes can seed bone marrow of myeloablated mice and modulate atherosclerosis. In addition, our study shows the potential of splenocytes for therapeutic interventions in inflammatory disease.

Introduction

Current knowledge indicates that splenic hematopoiesis contributes to variety of inflammatory and degenerative diseases [1–10]. Splenic hematopoiesis has been reported in several animal

models of disease including cancer [11], atherosclerosis [12–14] and myocardial infarction [15]. These studies suggest that hematopoietic stem cells originating from the bone marrow accumulate in high numbers within the spleen of diseased animals and they become more skewed toward myelopoiesis at the expense of erythropoiesis and lymphopoiesis [11]. Whether cells produced in the spleen differ from those derived from the bone marrow remains unknown.

Atherosclerosis is an inflammatory disease induced by a lipid metabolic disturbance at sites of hemodynamic strain in the vasculature. The inflammatory response manifests itself as infiltration and activation of mononuclear cells, activation of humoral cascade systems, cytokine secretion, cell death, and induction of fibroproliferative repair processes [16,17]. Innate immunity, which is represented by macrophages, is necessary for the initiation of atherosclerosis [18].

Macrophages are remarkably plastic and it is believed that they can change their functional phenotype depending on the environmental cues [19]. Similar to the T helper type 1 and T helper type 2 polarization, two distinct states of polarized activation for macrophages have been defined: the classically activated (M1) macrophage phenotype and the alternatively activated (M2) macrophage phenotype [20,21]. In addition to external factors, macrophages respond to changes in their intracellular milieu, such as cholesterol loading by inducing highly specified adaptive mechanisms and reactions [22,23]. Recent evidence suggests that origin of tissue macrophages play a role in the determination of macrophage phenotype [24], suggesting that macrophages may have an intrinsic gene expression program that is tissue dependent.

We determined the ability of spleen cells to seed bone marrow and to reconstitute myeloid system of lethally-irradiated mice. In addition, we compared the atherogenic and atheroprotective activities of spleen cells with bone marrow cells.

Materials and Methods

Mouse Strains

All wild type and mutant mice were in C57BL/6 background. The wild type, GFP, and apo E-/- mice were purchased from Jackson laboratories (Maine, USA). All animal protocols approved by the Institutional Animal Care and Use committee at Cedars-Sinai Medical Center. All mice used in this study were 7–9 weeks of age and they were monitored daily. For mouse euthanasia, when the mice reached the described time points we used an isoflurane overdose and subsequent cervical dislocation to ensure death. For the radiation controls, since the mice were not transplanted with bone marrow yet they receive a lethal dose of radiation, they should die approximately 2 weeks post-irradiation. These mice were observed daily for signs that they are moribund. When this became apparent, they are euthanized described.

In some experiments, the animals were fed a high fat diet (Harlan) as indicated starting at 4 weeks after spleen cell or bone marrow cell transplantation, with water taken ad libitum. The blood samples were collected at the indicated times, the plasma was separated from the red blood cells by low speed centrifugation (2000 rpm in a microcentrifgue for 20 mins) and the plasma cholesterol was quantified by a kit from Wako Chemicals (Virginia, USA) essentially as described by manufacturer. After the indicated times of being fed an atherogenic diet, terminal blood samples were collected by puncture of the right ventricle following euthanasia by an isoflurane overdose. Mice were perfused with PBS (20 ml.) via the left ventricle, while the perfusate drained from the punctured right atria. The heart and ascending aorta to the iliac bifurcation were removed for analysis.

Splenocyte Transplantation

For splenocyte transplantation, female recipient mice were lethally-irradiated followed by the infusion of adult splenocytes from donor male mice, essentially as previously described for

bone marrow transplantation [25,26]. Briefly, spleen cells were harvested from 4- to 5-week old donor male mice and injected into the tail vein of lethally-irradiated female recipient mice (5- to 7-weeks old, $1x10^7$ cells/mouse). For bone marrow transplantation, bone marrow was harvested from the femurs and tibias of male donor mice and injected into the tail vein of lethally-irradiated female recipient mice ($1x10^7$ cells/mouse). To ensure that the endogenous myeloid cells are completely ablated, we determined the radiation dose that completely ablated myeloid system of the recipient mouse. All the irradiated mice (n = 5) that were not transplanted with any donor cells died within one week of irradiation. For comparison, bone marrow transplantation was performed utilizing bone marrow from the same donor mouse that was used for splenocyte transplantation.

The donor cells were derived either from GFP mice or apo E-/- mice, depending on the experiments. The recipient mice were wild type C57BL/6 mice or apo E-/- mice, depending on the experiments. The presence of GFP facilitates analysis of engrafted cells. As another measure of engraftment efficiency, we analyzed Y chromosome in the recipient mice using PCR primers specific for the Y chromosome (see S1 Fig for analysis).

All the recipient mice were found to be healthy, physically active, and had similar body weight. For atherosclerosis studies, the recipient mice were fed an atherogenic diet 4 weeks after transplantation. At 16 weeks post atherogenic diet, mice were sacrificed, and their lesion size and phenotype were determined.

Flow Cytometry

The freshly harvested cells were analyzed by flow cytometry essentially as described [27]. Briefly, red blood cells were removed following centrifugation by re-suspending the harvested cells in the 1X red blood cell lysis buffer for 5 min. The cells were centrifuged again, washed 3 X with PBS buffer, and the cell number was determined with Beckman-Coulter Z1 Automated Cell Counter. Cell viability was assessed using trypan blue exclusion and cell morphology was examined using light microscopy. For all experiments, cell suspensions were preincubated with anti-CD16/CD32 mAb (Biolegend) to block FcγRII/III receptors. Cell staining was performed in the dark for 30 min at 4°C in staining buffer. The reagents used for flow cytometry and the list of antibodies were shown in S1 Table and S2 Table, respectively. Cells were analyzed on Cedars-Sinai Flow Cytometry Core Facility instruments (Beckman-Coulter (Dako, CyAn). Data were analyzed with Summit software. The engrafted GFP^+ cells were re-gated for the expression of CD11b/F4/80 (monocytes/macrophages), Siglec-F (eosinophils), and Ly6G (neutrophils). For more information about the flow cytometry reagents see S1 Table and S2 Table.

Morphology and Histology

The histochemical and immunostaining were performed essentially as described [28–30]. Briefly, mice were euthanized by an isoflurane overdose and perfused with phosphate-buffered saline (PBS) through the left ventricle. Aortas were dissected from aortic arch to iliac bifurcation and fixed in 4% paraformaldehyde (PFA)-PBS overnight. Frozen sections were prepared and stained with anti-MOMA-2 (macrophages) and smooth muscle -α-actin (smooth muscle cells) antibodies essentially as described [25,30,31]. Standard Trichrome staining was used for collagen. For quantification of the slides, we used the Image-Pro Plus software (Media Cybernetics, Inc., Bethesda, MD) under 400x magnification. Four high-power fields were used for each segment. Microscope lighting, focus, and field selection were optimized for distinction of cell/stain boundaries.

Atherosclerotic Lesion Analysis

Four weeks after transplantation, the recipient mice were fed an atherogenic diet for 16 weeks, after which they were sacrificed, and their lesion size and phenotype were determined. The extent of the atherosclerotic lesions in the Oil red O-stained sections was quantified, as described previously [30,32]. For aortic sinus analysis, tissue samples were snap frozen in liquid nitrogen and cut as 10 μm frozen sections. Cross-sectional lesion areas were quantified from aortic roots stained with Hematoxylin-Eosin. The percentage of the lesion area from intima was calculated from a tissue section of aortic sinus level, defined by the presence of three valve cusps. For aortic lesion analysis, regions of each aorta quantified were defined as follows: (1) thorax, from the arch to the intercostal artery branch; and (2) abdominal region, from the thorax to the branch of the iliac bifurcation. Aortas were opened longitudinally and attached to a black surface. Aortas were stained with Oil red O (Sigma-Aldrich) and photographed for en face evaluation. The lesion size for each aorta was measured by Image-Pro Plus (version 4, Media Cybernetics). Lesions were reported as percentage of the total aortic area consisting of thoracic aorta (ending at the final intercostal artery), and abdominal aorta (ending at the iliac bifurcation).

Macrophage Cell Culture

Bone marrow cells or splenocytes were cultured in the presence of L929-cell conditioned medium as a source of colony stimulating factor [33]. The cells were cultured in RPMI1640 supplemented with 20% fetal bovine serum (Gibco, cat. 12657–029), 30% L929 cultured media, 100 U/ml penicillin, 100 μg/ml streptomycin, and 2 mM L-glutamine. Cells were seeded in tissue culture treated dishes (BD Biosciences) and incubated at 37°C.

To culture spleen macrophages under the condition that is similar to those derived from bone marrow, we seeded 6X more spleen cells than bone marrow cells into culture dishes because the number of hematopoietic stem cells in the spleen was 6X lower than those in bone marrow. Despite this, the cultured bone marrow-derived macrophages became confluent at 5 days whereas spleen-derived macrophages required 12 days to reach the same degree of confluency.

qPCR Analysis

Total RNA extraction and mRNA expression analysis by real-time quantitative reverse-transcription polymerase chain reaction (qPCR) were performed as described previously [29,30]. The mRNA expression levels of all genes were normalized to the reference gene glyceraldehyde 3-phosphate dehydrogenase (GAPDH). The primer sequences are summarized below. For information about the primers see S3 Table.

Statistical Analysis

Statistical analysis was performed with GraphPad Prism Software (version 6.0.4, GraphPad, La Jolla, California). All values are presented as mean±SEM. One-way analysis of variance followed by posthoc Bonferroni correction for multiple comparisons. A p value <0.05 was considered statistically significant.

Results

Splenocyte Transplantation

To explore the ability of splenocytes to repopulate bone marrow of myeloablated mice, we used GFP^+ mice as donors because it facilitates the analysis of engrafted cells (S1 Fig). The phenotypes of donor splenocytes and bone marrow cells are shown in S2 Fig. After transplantation of

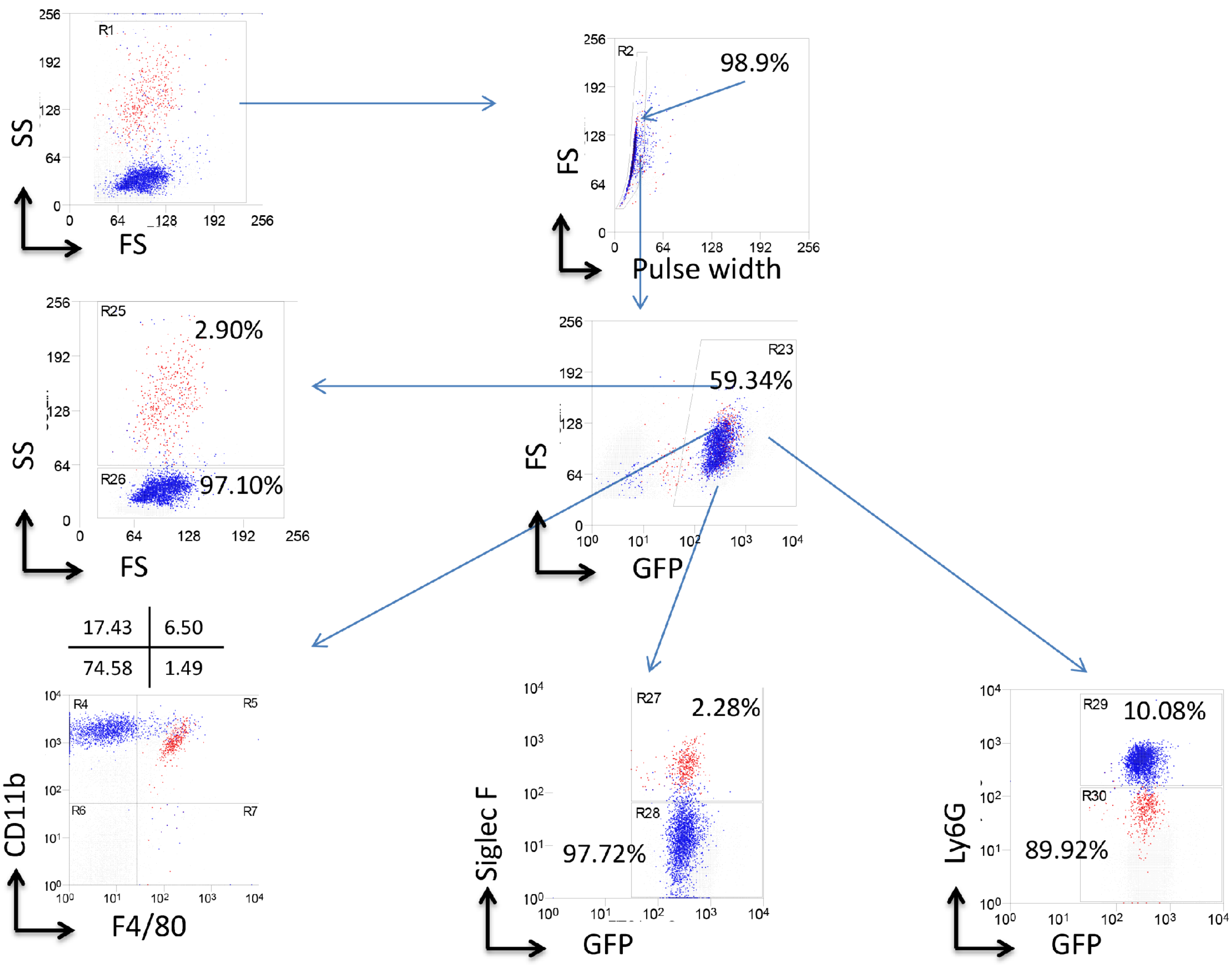

Fig 1. Flow cytometry gating strategy is shown that was used to analyze spleen and bone marrow from wild type recipient mice that were transplanted with donor cells from GFP+ mice. Cells were harvested as described in the Method section.

doi:10.1371/journal.pone.0125961.g001

donor splenocytes and bone marrow cells into recipient mice, the phenotypes of engrafted cells were determined by flow cytometry. The flow cytometry strategy is outlined in Fig 1. GFP$^+$ splenocytes and bone marrow cells originating from the donor mice were found in the spleen and bone marrow of recipient mice. The level of engrafted GFP$^+$ cells in the spleen of recipient mice was similar whether the donor cells were originated from spleen or bone marrow (Fig 2, left panel). Similarly, no significant difference in the level of engrafted GFP$^+$ cells was detected in the bone marrow of recipients that were transplanted with either donor splenocytes or bone marrow cells (Fig 2, right panel). Despite this similarity in the level of engraftment of donor cells, the overall level of engrafted GFP$^+$ cells in the recipient bone marrow was significantly higher than those in the recipient spleen (Fig 2).

To determine the phenotype of engrafted GFP$^+$ cells, the linage of the recipient cells were analyzed by flow cytometry. We found that the donor cells gave rise to macrophages (Fig 3); however, the levels of GFP$^+$-macrophages present in either the recipient's bone marrow or

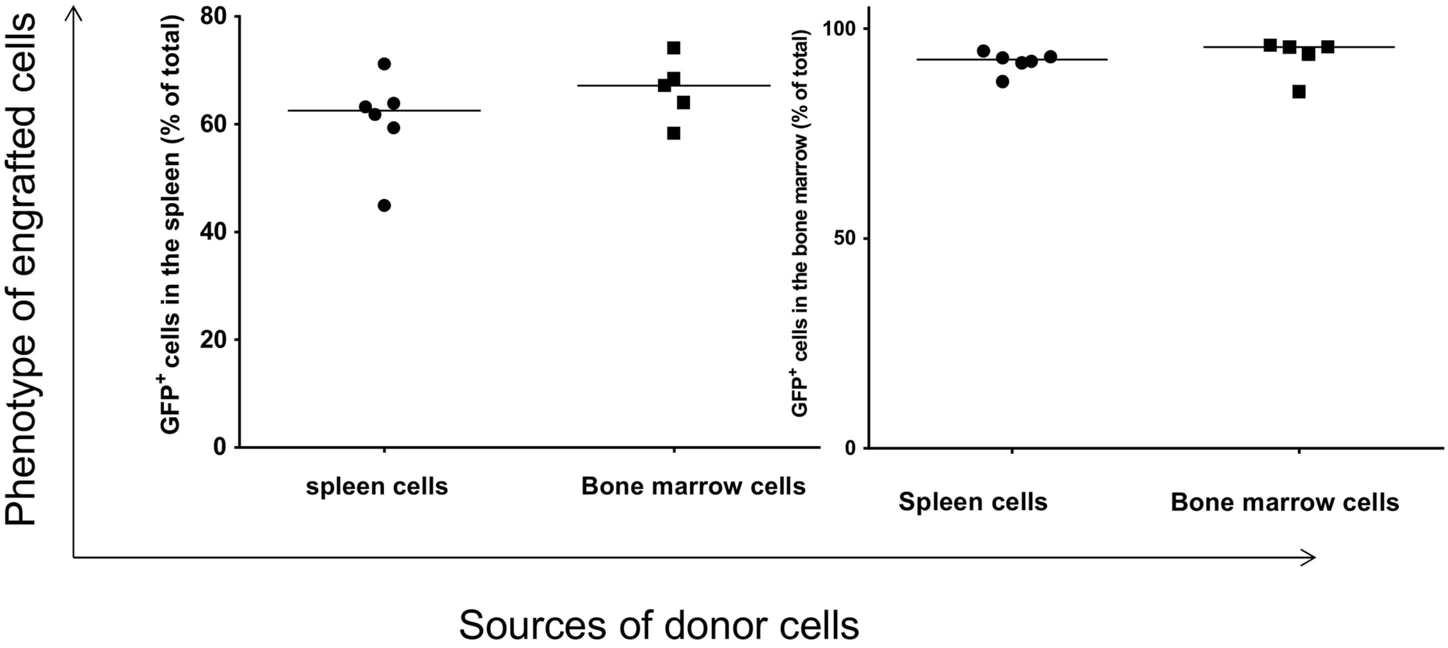

Fig 2. Flow cytometric analysis of spleen cells and bone marrow from recipient mice that were transplanted with donor spleen or bone marrow cells. Each data point represents one mouse.

doi:10.1371/journal.pone.0125961.g002

their spleen is dependent on the origin of donor cells; i.e., the level of GFP+-macrophages in the recipient spleen is higher when the donor cells were originated from bone marrow than from spleen ($p = 0.02$). Conversely, the level of GFP+-macrophages in the recipient's bone

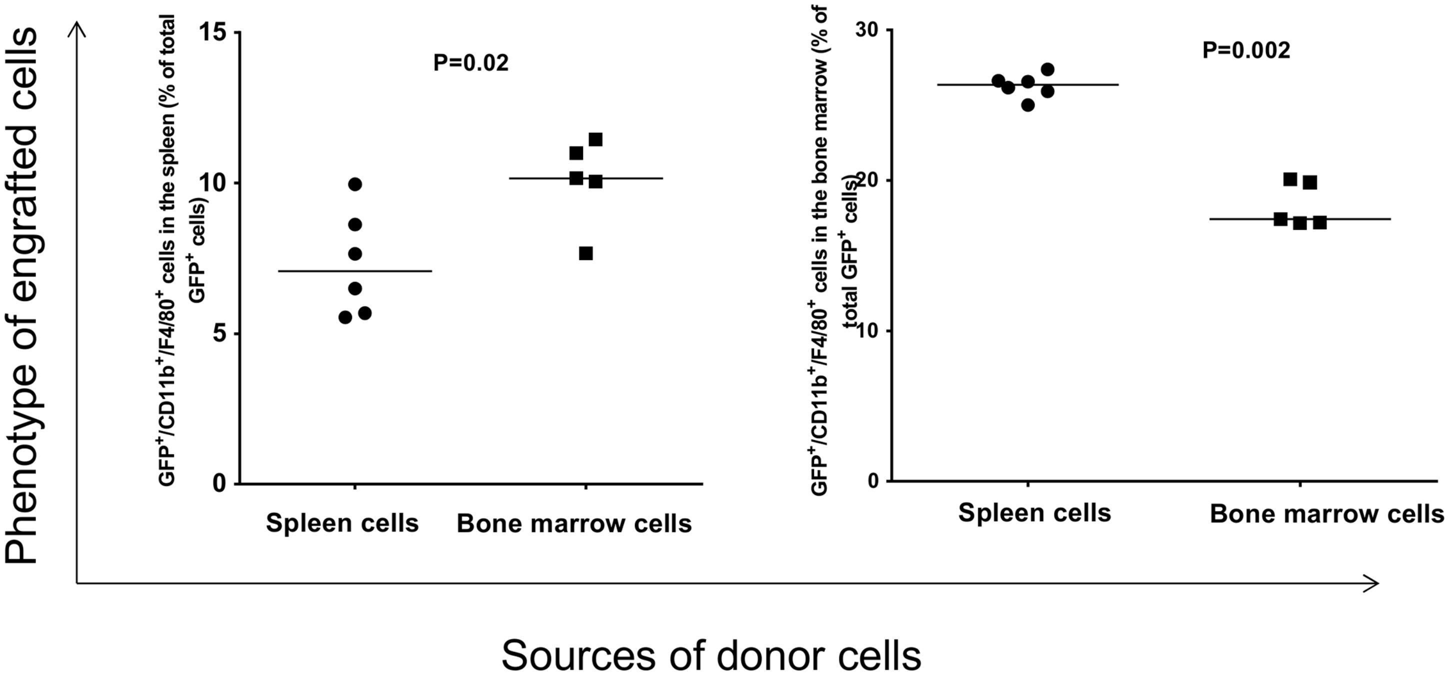

Fig 3. Analysis of engrafted GFP+ cells in the spleen and bone marrow of recipients that gave rise to macrophages. Each data point represents one mouse.

doi:10.1371/journal.pone.0125961.g003

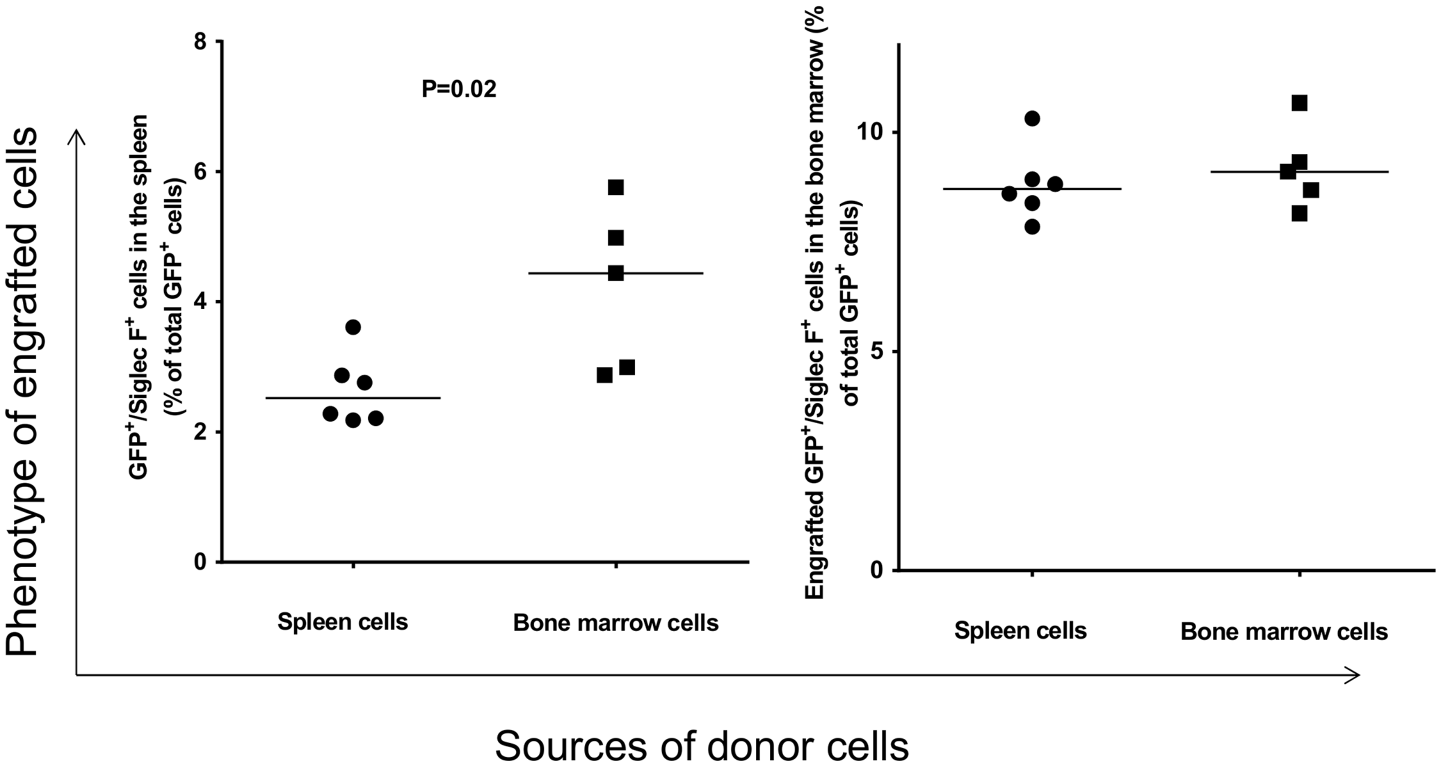

Fig 4. Analysis of engrafted GFP$^+$ cells in the spleen and bone marrow of recipients that gave rise to eosinophils. Each data point represents one mouse.

doi:10.1371/journal.pone.0125961.g004

marrow was significantly higher when the cells originated from spleen (p = 0.002). Thus, there are differences in the ability of splenocytes and bone marrow to differentiate into macrophages in the spleen and bone marrow of recipient mice.

Analysis of GFP$^+$ cells for differentiation toward an eosinophil linage revealed that the donor splenocytes and bone marrow cells gave rise to eosinophils in the spleen and bone marrow of recipient mice (Fig 4). However, the ability of donor splenocytes to differentiate into eosinophils is significantly lower than those of donor bone marrow cells in the spleen of recipient (p = 0.02). No such difference was detected in the bone marrow of recipient (Fig 4, right panel).

Similar results were obtained when splenocytes and bone marrow cells isolated from recipient mice were analyzed for the presence of GFP$^+$/Ly6G$^+$ neutrophils (Fig 5). In the spleen of recipient, the level of donor GFP$^+$/Ly6G$^+$ cells is significantly higher when the donor cells were originated from bone marrow than those that originated from splenocytes (Fig 5, left panel, p = 0.002). In the bone marrow of recipient, we did not detect such differences (Fig 5, right panel).

Next we asked whether these differences in the ability of splenocytes and bone marrow cells to differentiate into different myeloid cell linages are related to hematopoietic stem cells (LSK). To explore this, we analyzed the frequency of LSK cells in the spleen and bone marrow of the wild type mice. The lineage negative cells (GFP$^-$/Lin$^-$) were re-gated for the expression of c-Kit and Sca-1. No staining and isotype-specific staining were used to control for autofluorescence and non-specific staining, respectively (Fig 6). The analysis of these cells in the bone marrow of recipient mice showed that the level of LSK cells in the bone marrow of recipient mice is

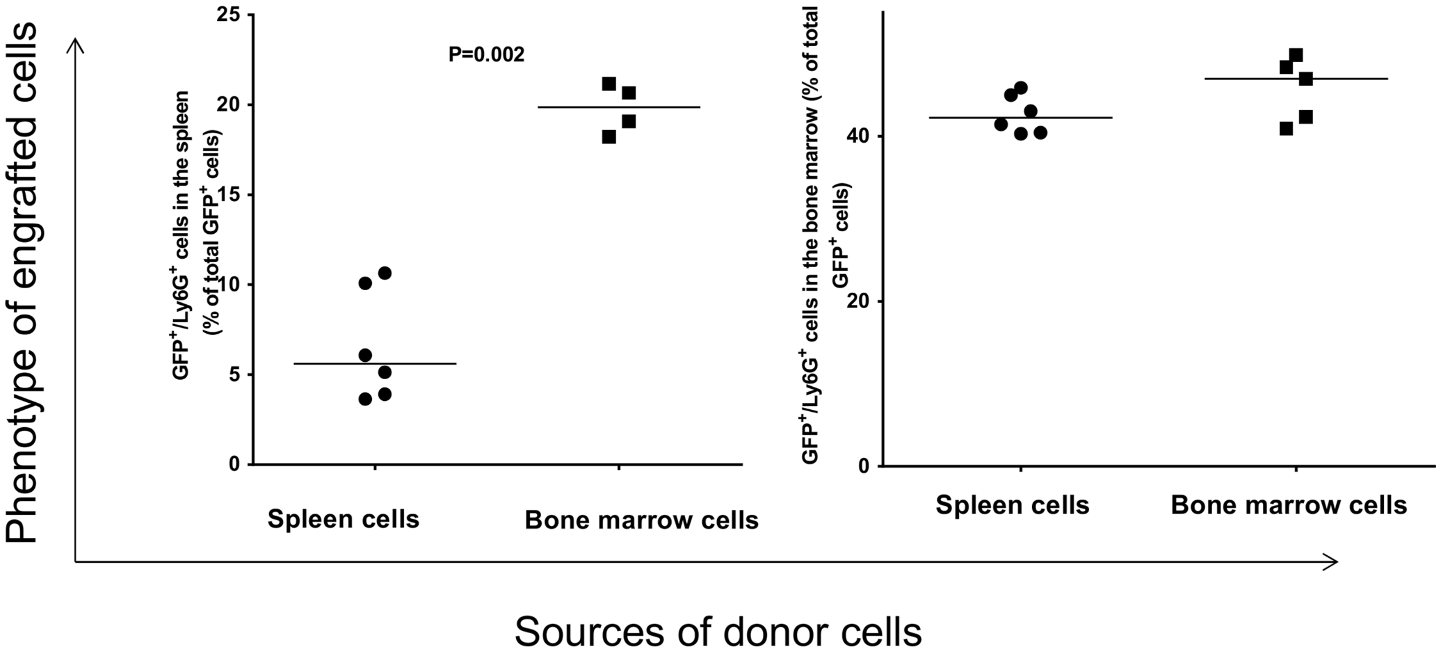

Fig 5. Analysis of engrafted GFP$^+$ cells in the spleen and bone marrow of recipients that gave rise to neutrophils. Each data point represents one mouse.

doi:10.1371/journal.pone.0125961.g005

significantly higher than those in the spleen (Fig 7). Thus, while the level of hematopoietic stem cells is significantly lower in the donor spleen than in the donor bone marrow; this difference does not correlate with the level of macrophages that were originated from spleen as compared to bone marrow.

Atherosclerosis Studies

To determine whether differences in the level of macrophages and neutrophils in the recipient bone marrow and spleen have a pathophysiological significance, we performed atherosclerosis studies. We chose this mouse model because both macrophages and neutrophils contribute to the pathogenesis of atherosclerosis [34,35]. We performed two studies: first, the effect of splenocyte transplantation on the progression of atherosclerosis was investigated; in the second study, the ability of splenocytes to rescue an atherogenic phenotype of apo E-/- mice was examined. For the progression study, female recipient mice were lethally-irradiated followed by the infusion of adult splenocytes from donor male apo E-/- mice, essentially as previously described for bone marrow transplantation [25,26]. For the rescue study, donor cells harvested from GFP$^+$ male mice were transplanted into female apo E-/- recipient mice. It is important to note that we did not use any helper bone marrow cells for transplantation of splenocytes; thus, the splenocytes-mediated reconstitution is entirely dependent on the donor adult spleen cells.

In the progression studies (Fig 8, left panels), we found significant lesions in the aortic sinuses of apo E-/- recipient groups that were transplanted with either splenocytes (Fig 8, top left panel) or their bone marrow cells (lower left panel). The lesions were complex, featuring a lipid core and cholesterol clefts. Some lesions were calcified. In the rescue experiments (Fig 8, right panels), transplantation of either splenocytes (top right panel) or bone marrow cells (lower right panel) from GFP$^+$ donor mice markedly reduced the size of lesions.

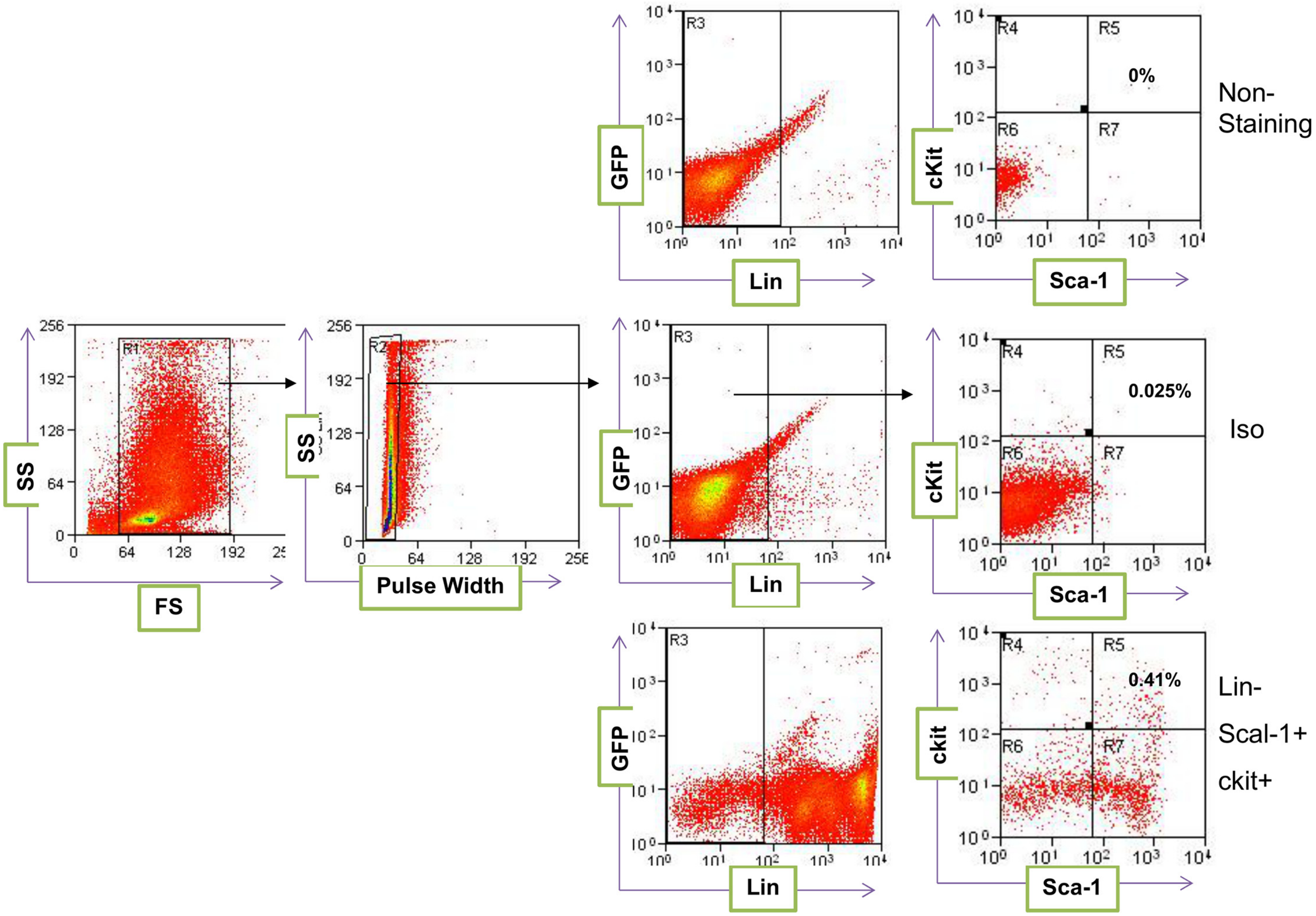

Fig 6. Representative flow cytometry gating strategy of bone marrow cells. Analysis of hematopoietic stem cells in the spleen and bone marrow of wild type C57BL/6 mice. The single cell population from wild type C57BL/6 donor mice was gated for lineage negative (Lin$^-$) and GFP$^+$ cells. The Lin$^-$/GFP$^+$ cells were re-gated for the expression of c-Kit and Sca-1. The nonstained cells and isotype control antibodies were used as negative controls. Each data point represents one mouse.

doi:10.1371/journal.pone.0125961.g006

Quantification of the lesions showed that transplantation of splenocytes from GFP donor mice into apo E-/- recipients markedly reduced lesion size when compared with the control apo E-/- donor mice, 0.99±0.02 mm2 vs. 0.2±0.01 mm2, respectively (Fig 9). Similarly, the transplantation of bone marrow cells from GFP$^+$ donors into apo E-/- recipients reduced the lesion size when compared with the control, 1.0±0.03 mm vs. 0.24±0.04 mm2 vs. respectively (Fig 9). No significant differences in the level of lesions were found in either study, suggesting that splenocytes display similar activity as those of bone marrow cells.

Effect of Splenocyte Transplantation on Serum Cholesterol

A previous report demonstrated that enhanced atherosclerosis in splenectomized rabbits was related to hyperlipidemia [36]. In addition, transplantation of bone marrow from wild type into apo E-/- mice has been shown to reduce hypercholesterolemia [37–40]. Therefore, we asked whether transplantation of splenocytes affects hyperlipidemia in the recipient mice. To

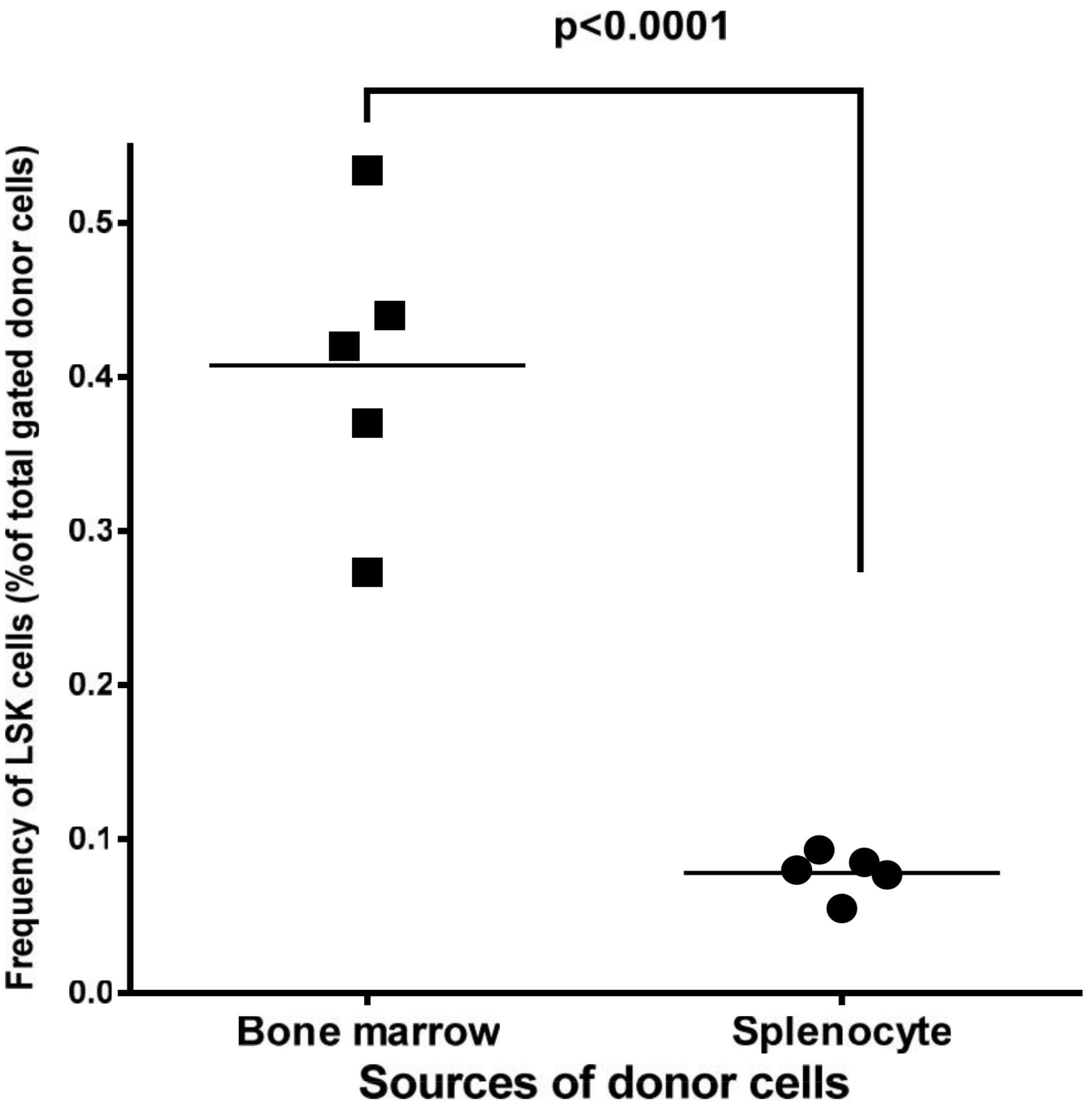

Fig 7. The quantitative analysis of hematopoietic stem cells (LSK) in the spleen and bone marrow of donor mice. Each data point represents one mouse.

doi:10.1371/journal.pone.0125961.g007

explore this, we measured the serum cholesterol level in the recipient mouse groups before and after high fat diet feeding. Before the atherogenic diet feeding, the serum cholesterol level in the GFP^+ donor mice was 78.0 ± 2.3 mg/ dl (Fig 10). The serum cholesterol level in the apo E-/- recipient cohorts that were transplanted with either splenocytes or bone marrow from apo E-/- donor mice was 367.9 ± 29.8 mg/dl and 421.8 ± 26.7 mg/dl, respectively (Fig 10). Transplantation of splenocytes or bone marrow cells from GFP^+ donor mice into apo E-/- recipient mice significantly reduced serum cholesterol levels to 87.3 ± 5.8 mg/dl and 92.7 ± 3.6 mg/dl, respectively (Fig 10). Thus, we found no significant difference in the serum cholesterol levels between the two recipient groups.

After feeding an atherogenic diet for 16 weeks, the serum cholesterol levels in the GFP^+ mice remained unchanged (Fig 11). However, in the apo E-/- donor/recipient groups, the serum cholesterol levels increased to 1424 ± 65.5 mg/dl and 1304 ± 72.5 mg/dl in the recipient of splenocytes and bone marrow, respectively. Transplantation of cells from GFP donor mice

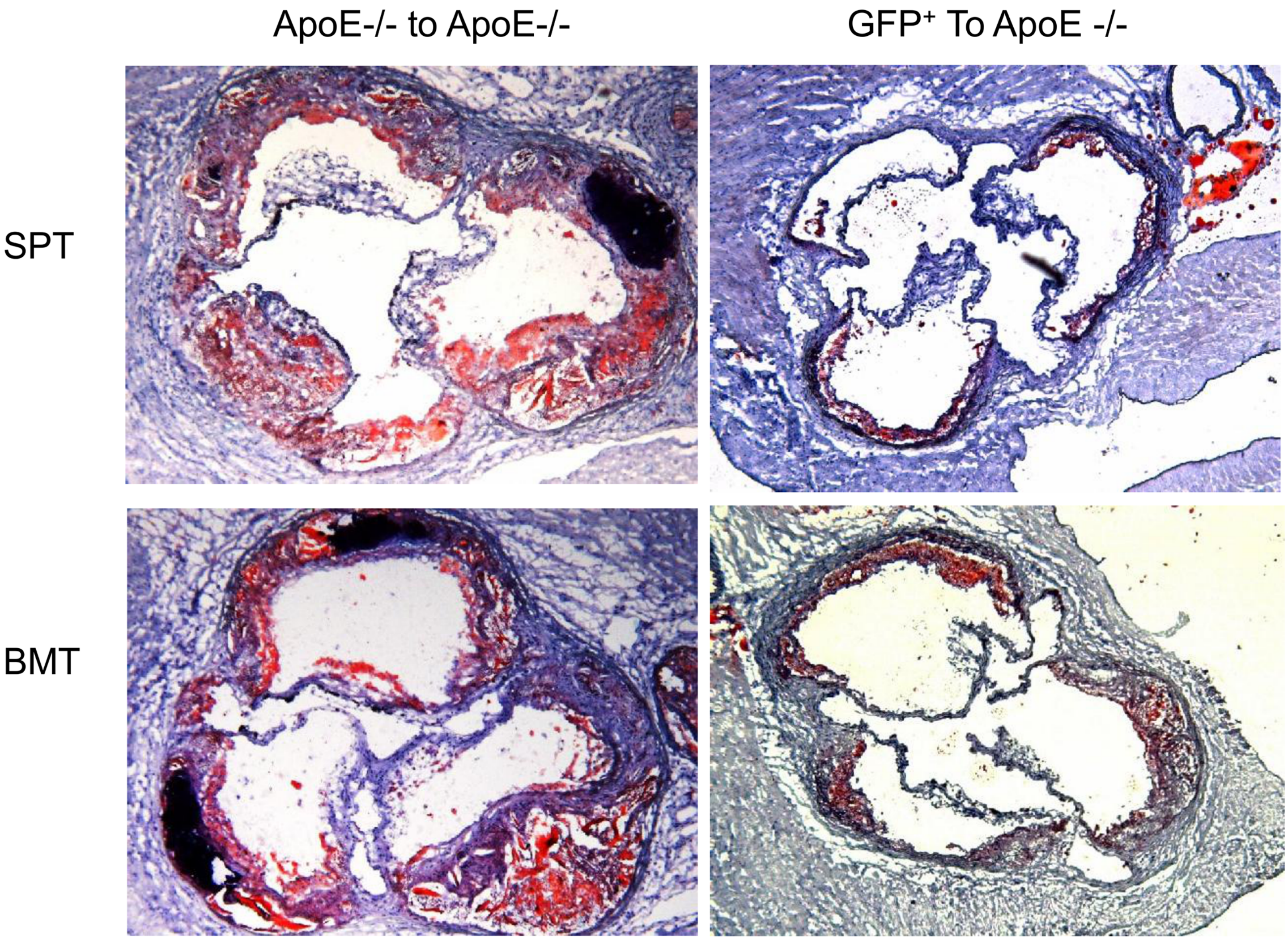

Fig 8. Aortic sinus lesions lipid staining in the apo E-/- recipient mice transplanted with donor cells. Apo E-/- mice (n = 10/group) were transplanted with splenocytes (left upper panel) or bone marrow cells (left lower panel) from apo E-/- donor mice. The right panels show aortic sinus lesions in the apo E-/- recipient mice transplanted with splenocytes (right upper panel) or bone marrow cells (right lower panel) from GFP^+ donor mice.

doi:10.1371/journal.pone.0125961.g008

into apo E-/- recipient reduced serum cholesterol to 254.2 ± 40.7 mg/dl and 221.2 ± 29.0 mg/dl in the recipient of splenocytes and bone marrow, respectively. No statistically significant difference in the serum cholesterol level was found between the two GFP^+ recipient groups.

Effect of Splenocyte Transplantation on the Phenotype of Plaques

To determine the effect of transplantation on the phenotype of lesions, we analyzed the lesions in the two groups of recipient mice for macrophages (Fig 12), smooth muscle cells (Fig 13) and collagen content (Fig 14). In the apo E-/- donor/recipient groups (Fig 12, left panels) macrophage were found to be primarily accumulated around the lipid core of aortic sinus lesions of recipient mice that were transplanted with either splenocytes (top left panel) or bone marrow cells ((lower left panel). The fibrous cap also contained significant numbers of macrophages. Transplantation of cells from GFP donor mice significantly reduced the level of macrophages in either donor splenocyte (top right panel) or donor bone marrow (lower right panel).

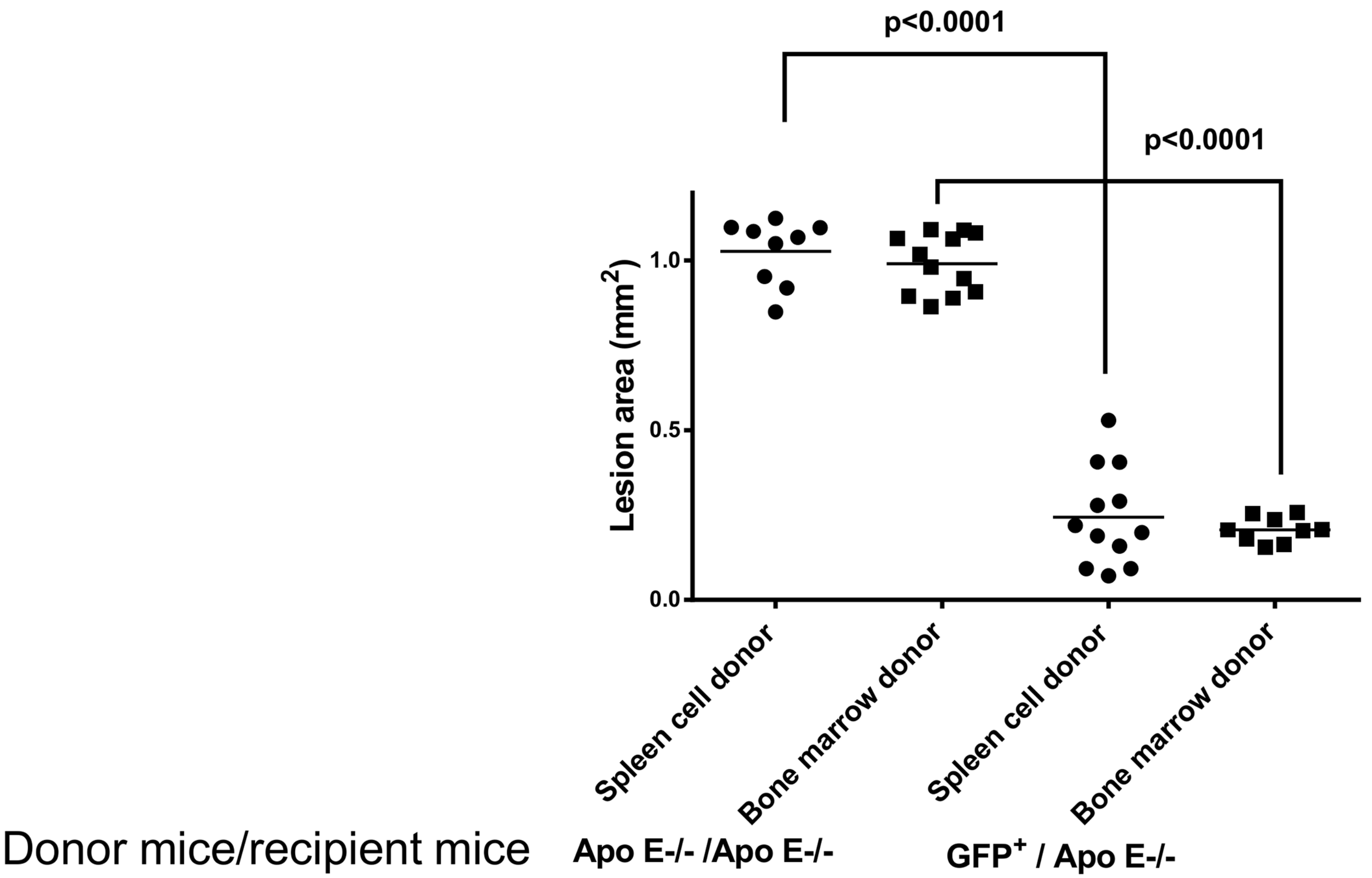

Fig 9. Quantitative analysis of lesion area in each recipient group shown in Fig 8.

doi:10.1371/journal.pone.0125961.g009

Analysis of lesions for smooth muscle cell content in the apo E-/- donor/recipient mice cohort showed that the staining was largely concentrated in the fibrous cap region with no obvious differences between the recipients of either splenocytes or bone marrow cells (Fig 13, left panels). Transplantation of donor cells from GFP^+ mice reduced lesions in the apo E-/- recipient mice and the smooth muscle cell staining were found primarily in the media (right panels). The collagen staining of the lesions showed that the fibrous cap of the lesions in both recipient groups transplanted with splenocytes or bone marrow cells from apo E-/- mice have significant lesions and the positive staining is primarily concentrated in the fibrous cap areas (Fig 14, left panels). In the recipient groups that were transplanted with either splenocytes or bone marrow cells from GFP^+ mice showed small lesions and collagen was largely concentrated in the media (right panels).

Quantitative analysis of lesions for macrophages (Fig 15), smooth muscle cells (Fig 16) and collagen content (Fig 17) in the recipient mice revealed no significant difference between the donor splenocytes and donor bone marrow cells. Thus, while there are differences in the ability of the two sources of donor cells to differentiate into myeloid cells in the bone marrow and spleen of the recipient mice, their atherogenic and atheroprotective activities were similar.

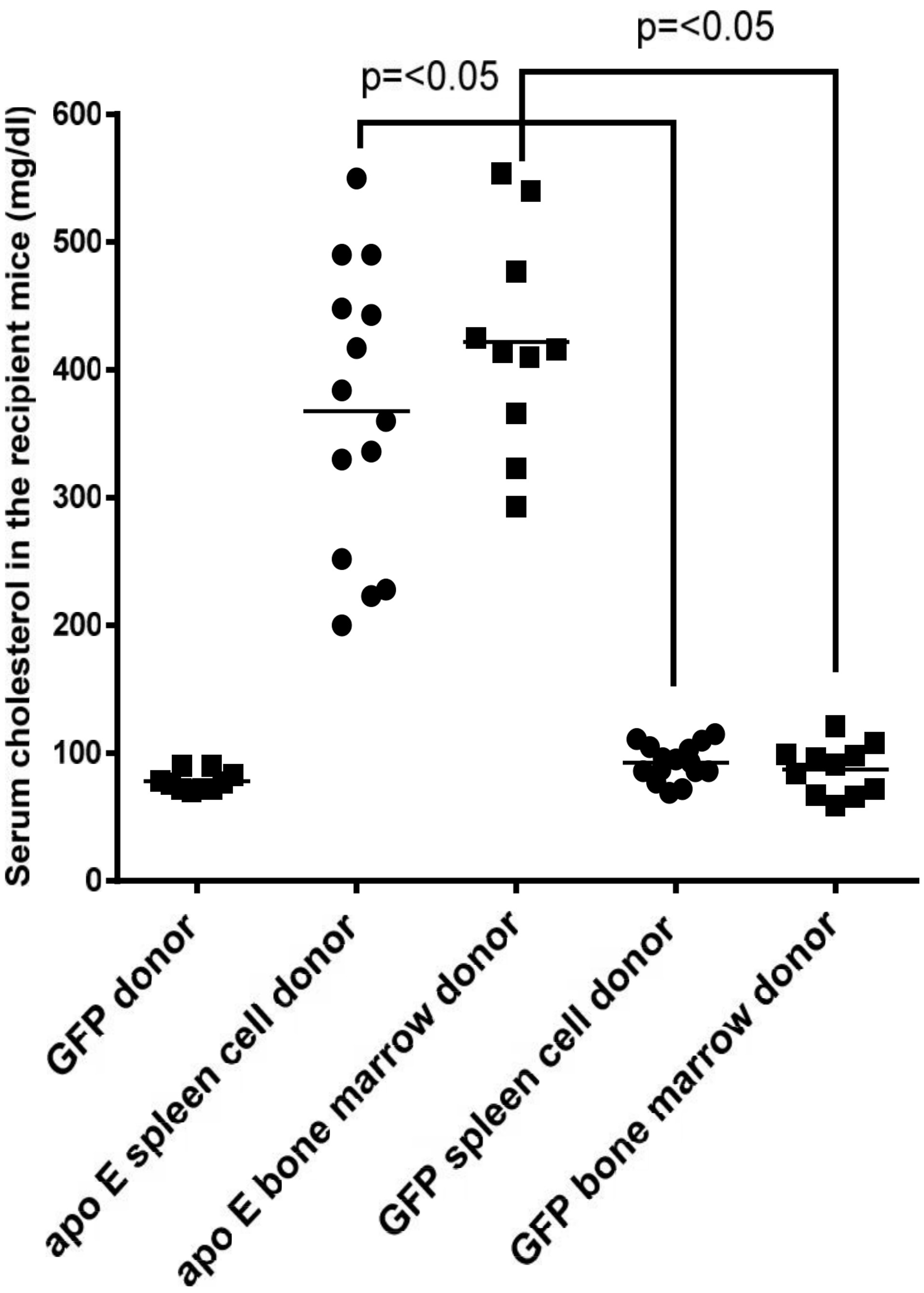

Fig 10. Shows the serum cholesterol levels of GFP+ donor mice and the recipient groups before high fat diet consumption. Each data point represents one mouse.

doi:10.1371/journal.pone.0125961.g010

Fate of Transplanted Splenocytes

Past studies have shown that apo E controls hematopoietic stem cells proliferation, monocytosis, neutrophilia, and monocyte accumulation in atherosclerotic lesions [12]. To determine the impact of apo E deletion on the lineage commitment of transplanted splenocytes, we analyzed the lineages of engrafted GFP+ donor cells in the bone marrow and spleen of lethally-irradiated

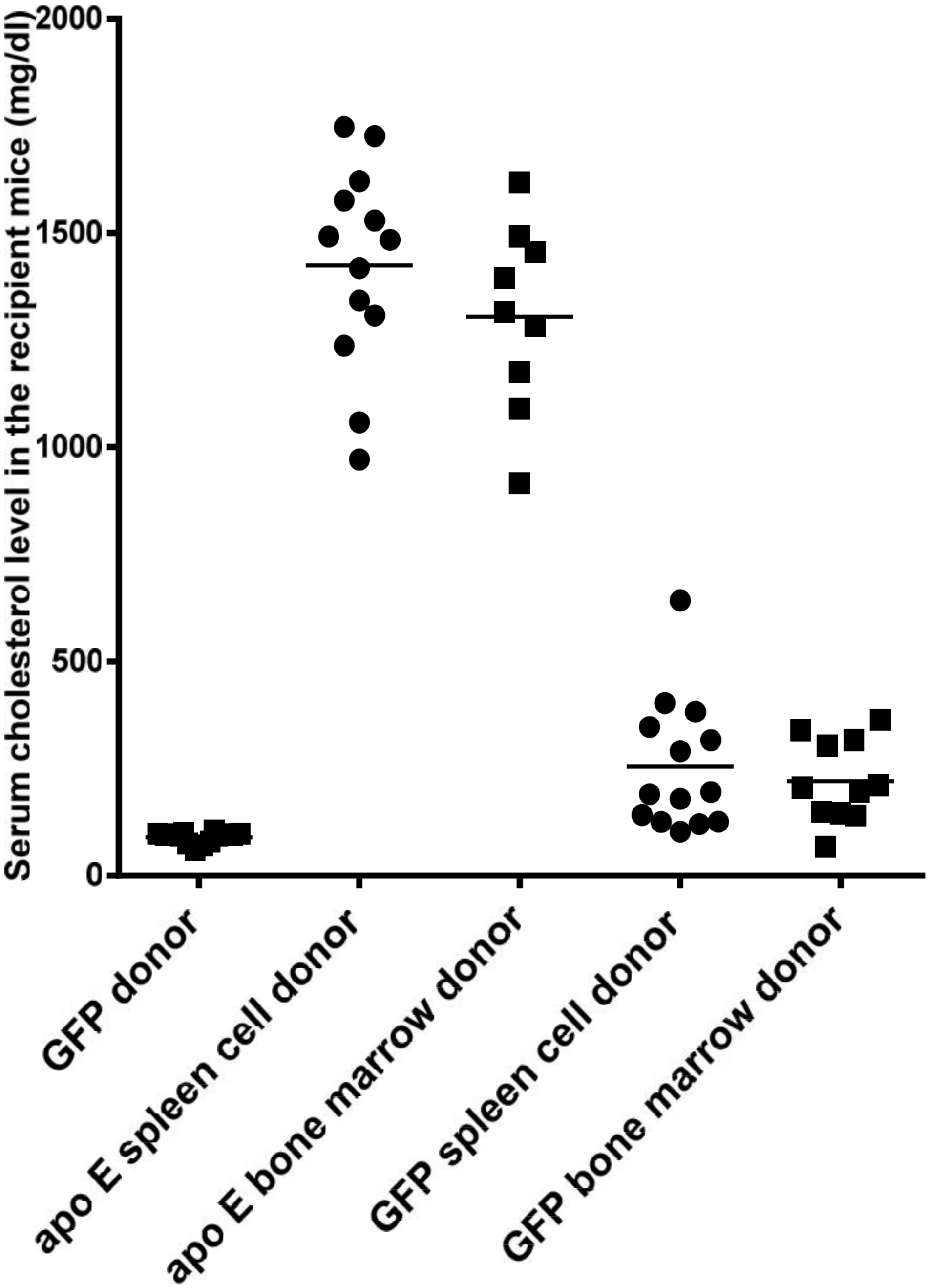

Fig 11. Shows the serum cholesterol levels of GFP$^+$ donor mice and the recipient groups after high fat diet consumption. Each data point represents one mouse.

doi:10.1371/journal.pone.0125961.g011

apo E-/- recipient mice. The splenocytes and bone marrow cells were isolated from apo E-/- recipient mice and analyzed for the expression of GFP followed by re-gaining of positive cells for macrophages, eosinophils, and neutrophils, essentially as described in Fig 1. The donor GFP$^+$ cells were successfully engrafted into apo E-/- recipient mice and the level of engraftment into spleen and bone marrow of recipient mice were similar irrespective of donor cell origin (Fig 18). The donor cells gave rise to macrophages (Fig 19) and neutrophils (Fig 20) in the bone

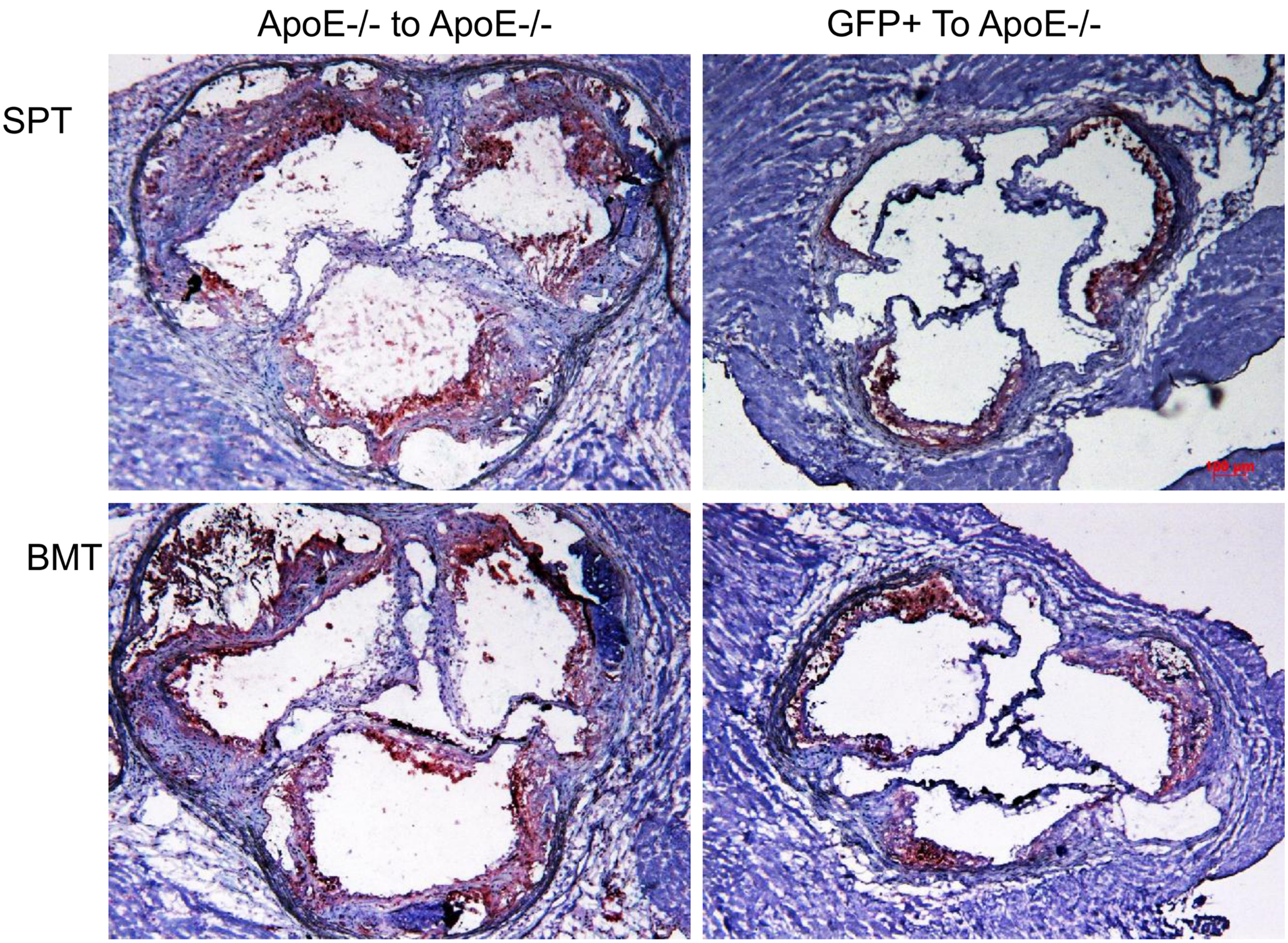

Fig 12. The aortic lesions in the recipient groups were stained for macrophages. The aortic sinus lesions in the apo E-/- donor/recipient groups (left panels) and GFP^+ donor/apo E-/- recipient groups (right panels) are shown.

doi:10.1371/journal.pone.0125961.g012

marrow and spleen of recipient mice and their levels were independent of the sources of donor cells. While the donor cells also gave rise into eosinophils (Fig 21); their level was higher when the cells originated from splenocytes rather than from bone marrow ($p = 0.02$).

Next, we analyzed the content of LSK cells in the apo E-/- recipient mice that were transplanted with either splenocytes or bone marrow cells before (Fig 22) and after (Fig 23) atherogenic diet feeding. The level of GFP^+/LSK cells in the bone marrow of recipient mice was similar between the two donor groups before feeding. However, analysis of recipient spleen revealed that the level of GFP^+/LSK is significantly higher when the bone marrow cells were used as the donor source as compared with splenocytes. Overall, the levels of donor LSK cells in the spleen of apo E-/- recipient mice was significantly lower than those in the bone marrow.

Similar level of LSK cells were detected in the recipient bone marrow and spleen after atherogenic diet feeding with one exception: the overall level of LSK cells in both spleen and bone marrow of recipient mice were reduced in response to atherogenic diet feeding when compared to normal diet (Fig 23). Thus, while there are significant differences in the frequency of LSK

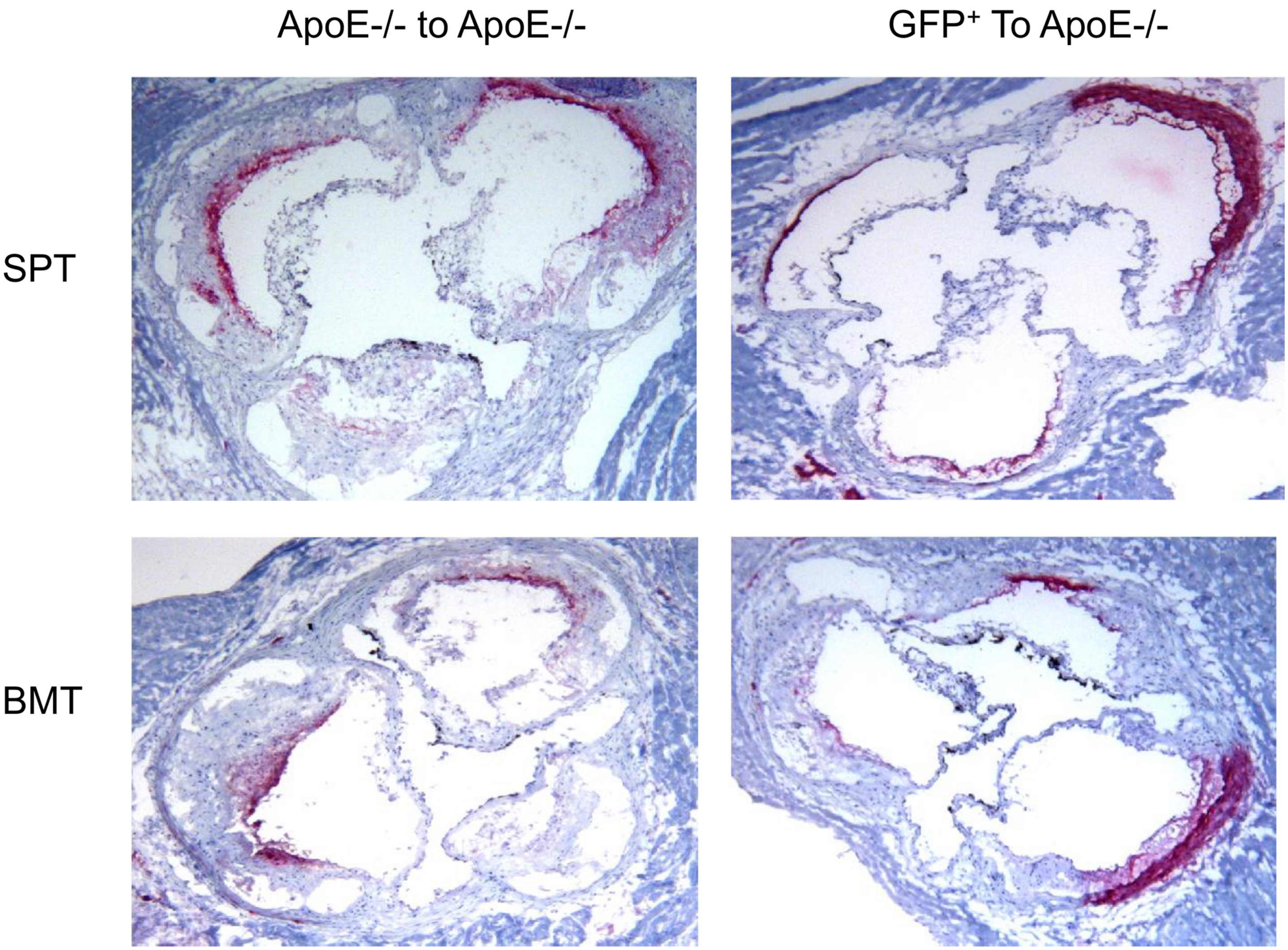

Fig 13. The same panel of sections as in Fig 12 were stained for α-actin (smooth muscle).

doi:10.1371/journal.pone.0125961.g013

cells in the spleen and bone marrow of recipient apo E-/- mice, these differences do not correlate with the level of differentiated myeloid cells in the spleen and bone marrow of recipient mice.

Phenotype of Cultured Macrophages

Macrophage phenotypes are thought to be regulated by their environment. Based on their ability to respond to Th1 vs Th2 cytokines, macrophages are classified as proinflammatory M1 or anti-inflammatory M2 subsets [19,41]. The IL-4/STAT6 axis has been recognized as a hallmark of M2 macrophage polarization whereas LPS/IFNγ/STAT1 signaling is responsible for M1 macrophage phenotype [42]. However, recent evidence suggests that some macrophage subsets may be "high wired" and intrinsic gene expression program also contribute to their diversity [43]. Understanding the contribution of extrinsic vs. intrinsic factors in the regulation of macrophage phenotype has important in vivo significance as dysregulation of macrophage function could lead to chronic inflammation and/or excessive fibrosis [44].

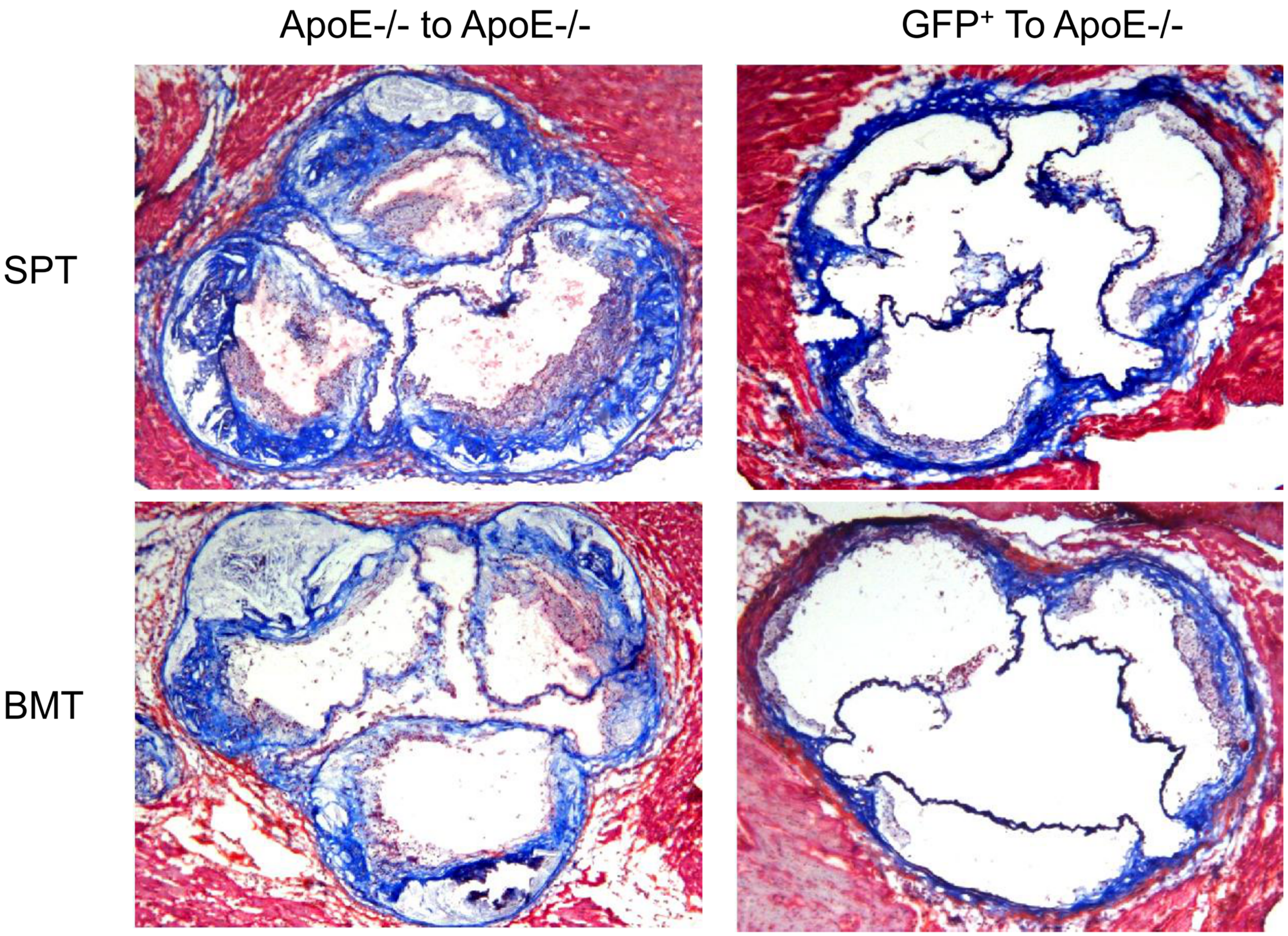

Fig 14. The same panel of sections as in Fig 12 were stained for collagen.

doi:10.1371/journal.pone.0125961.g014

To better understand the phenotype of macrophages in the spleen and bone marrow, we cultured spleen- and bone marrow-derived macrophages, treated them with LPS or IL-4, and the expression of M1/M2 markers were determined by qPCR. Analysis of M2 markers showed that the level of STAT6 in the untreated and IL-4-treated bone marrow-derived macrophages was markedly higher than that seen in spleen-derived macrophages (Fig 24). In contrast, the level of PPARγ in the two cell sources whether cells were untreated or treated with IL-4 were almost similar. The expression level of KLF2 was lower in the spleen-derived macrophages compared to bone marrow-derived macrophages under basal condition; however, the former cells were more responsive to IL-4 treatment than latter cells (Fig 24). The expression level of KLF4 was low in both cultured cells under basal conditions; however, bone marrow cells expressed markedly more KLF4 than spleen-derived macrophages in response to IL-4 treatment (Fig 24). The expression level of Arg-1 was low under basal conditions in the two cell types and its expression level was markedly induced in both cells to the same extent (Fig 25). In contrast, YM1 expression level was more than 60% higher in the bone marrow-derived macrophages than in spleen cells after treatment with IL-4 (Fig 25). With respect to M1 cell markers, the spleen-

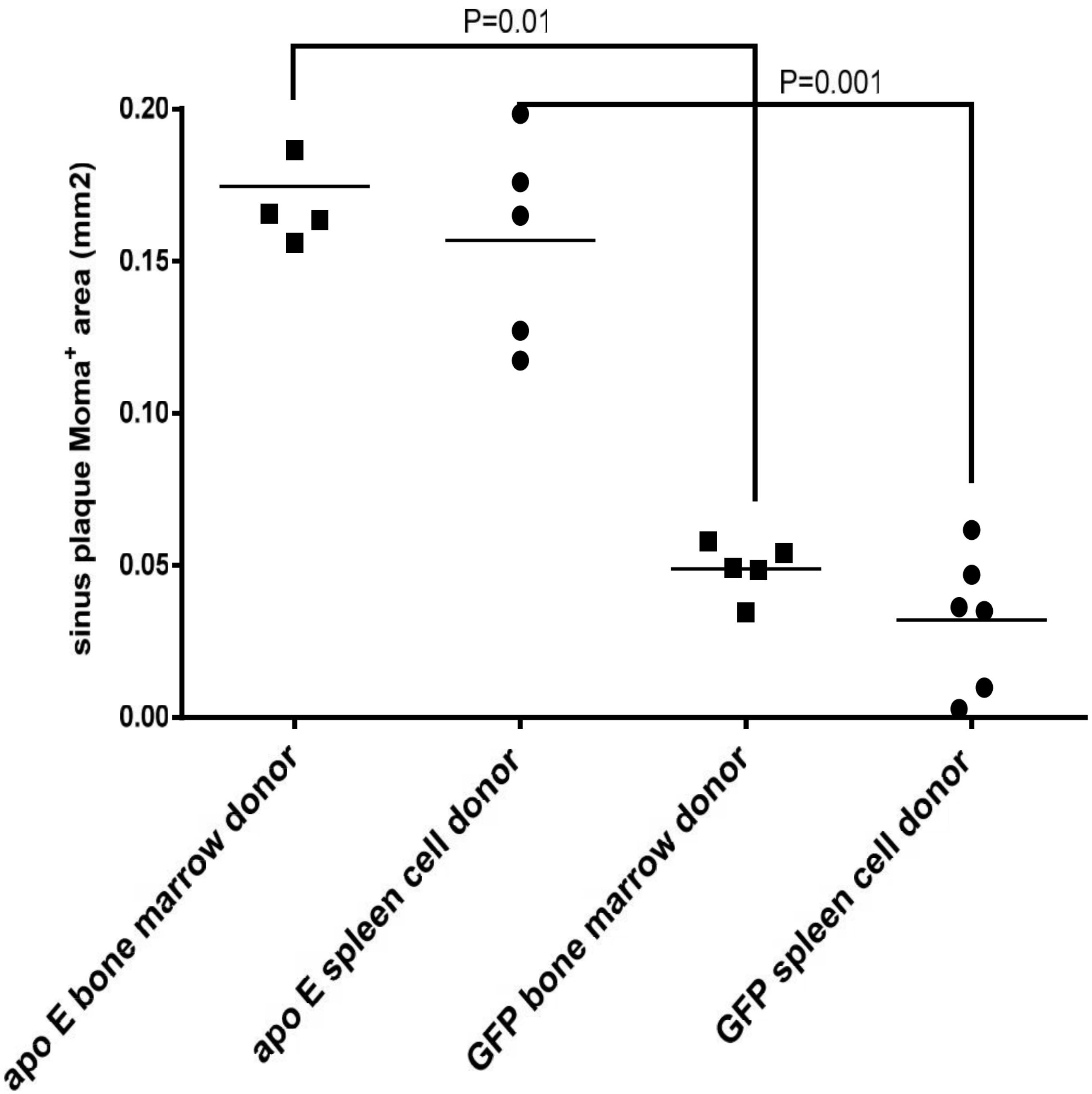

Fig 15. The quantification of lesion area for macrophage abundance as calculated using Moma-positive staining. See Method section for details.

doi:10.1371/journal.pone.0125961.g015

derived macrophages were more responsive than bone marrow cells in their expression of STAT1 in response to LPS (Fig 25). The expression level of MCP1 was markedly higher in the spleen derived macrophages than in bone marrow cells under basal conditions, and the expression of this gene was markedly induced after LPS treatment (Fig 26). The expression level of iNOS was low, in both cell types under basal conditions, and its expression level was markedly induced in both cell types (Fig 26). In the untreated cells, the expression level of TNFα was similar in both cultured cells; however, spleen derived-macrophages expressed a higher level of

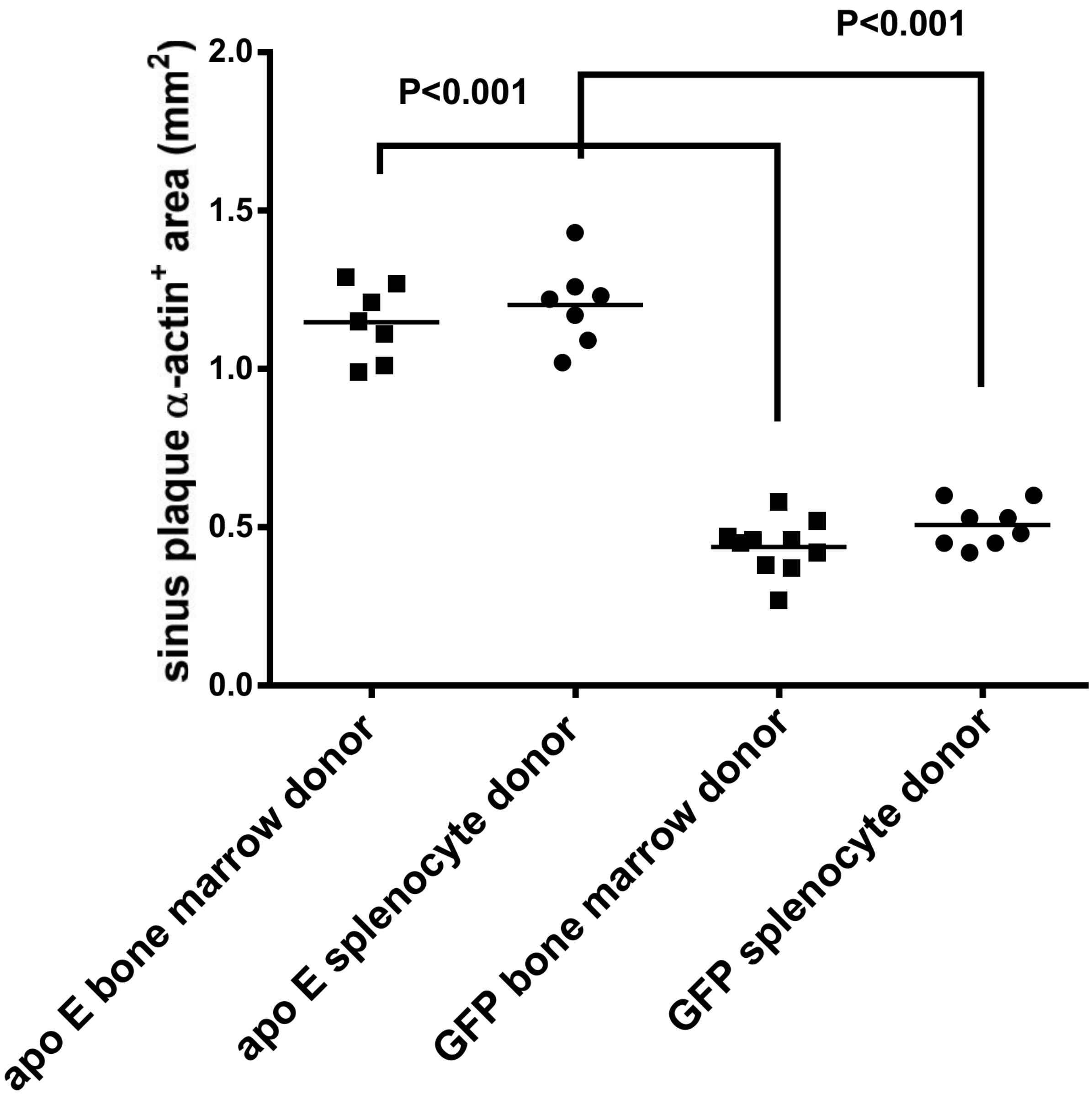

Fig 16. The quantification of lesion area for smooth muscle cells as determined by a positive staining with alpha actin. Please refer to method section for more details.

doi:10.1371/journal.pone.0125961.g016

TNFα than bone marrow macrophages in response to LPS treatment (Fig 26). In contrast, bone marrow-derived macrophages expressed markedly more COX2 in response to LPS when compared with spleen-derived macrophages (Fig 26). While freshly isolated splenocytes expressed Hox11 transcription factor, the cultured cells did not (data not shown), suggesting that the expression of Hox11 is an active process that can be induced by a spleen niche. Taken together, these data suggest that the spleen-derived macrophages have a stable phenotype that is different from those derived from bone marrow.

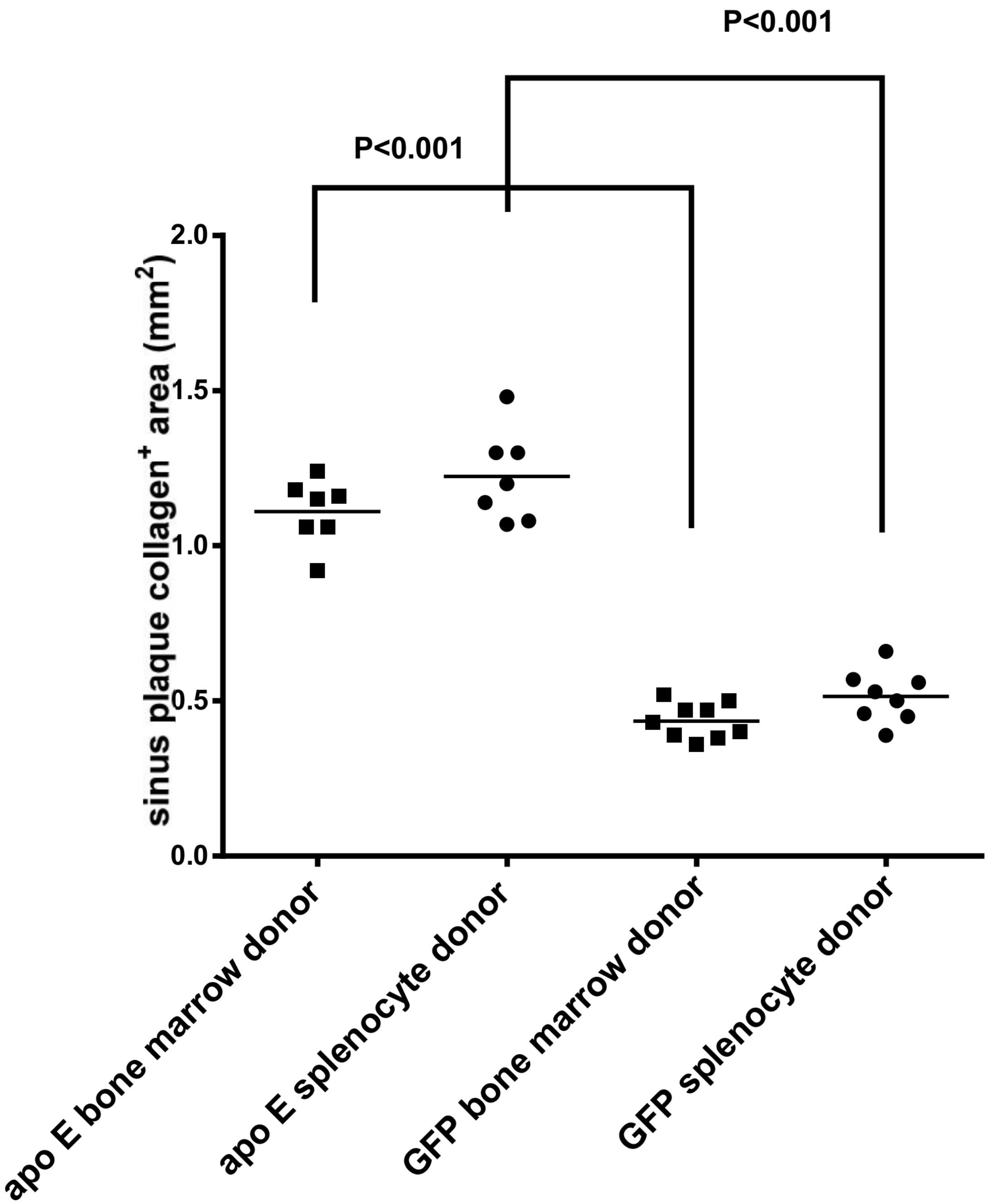

Fig 17. The quantification of lesion area for collagen contents was performed as described in the Method section.

doi:10.1371/journal.pone.0125961.g017

Discussion

In mammals and rodents, following birth, hematopoiesis occurs outside the medullary spaces of the bone marrow in a process called extramedullary hematopoiesis [45]. The liver and spleen are the main sites of extramedullary hematopoiesis; specifically, during inflammation, extramedullary hematopoiesis generates sufficient numbers of mature myeloid cells [46]. However, it is unclear whether this seeding process is a bidirectional event where splenocytes contribute to

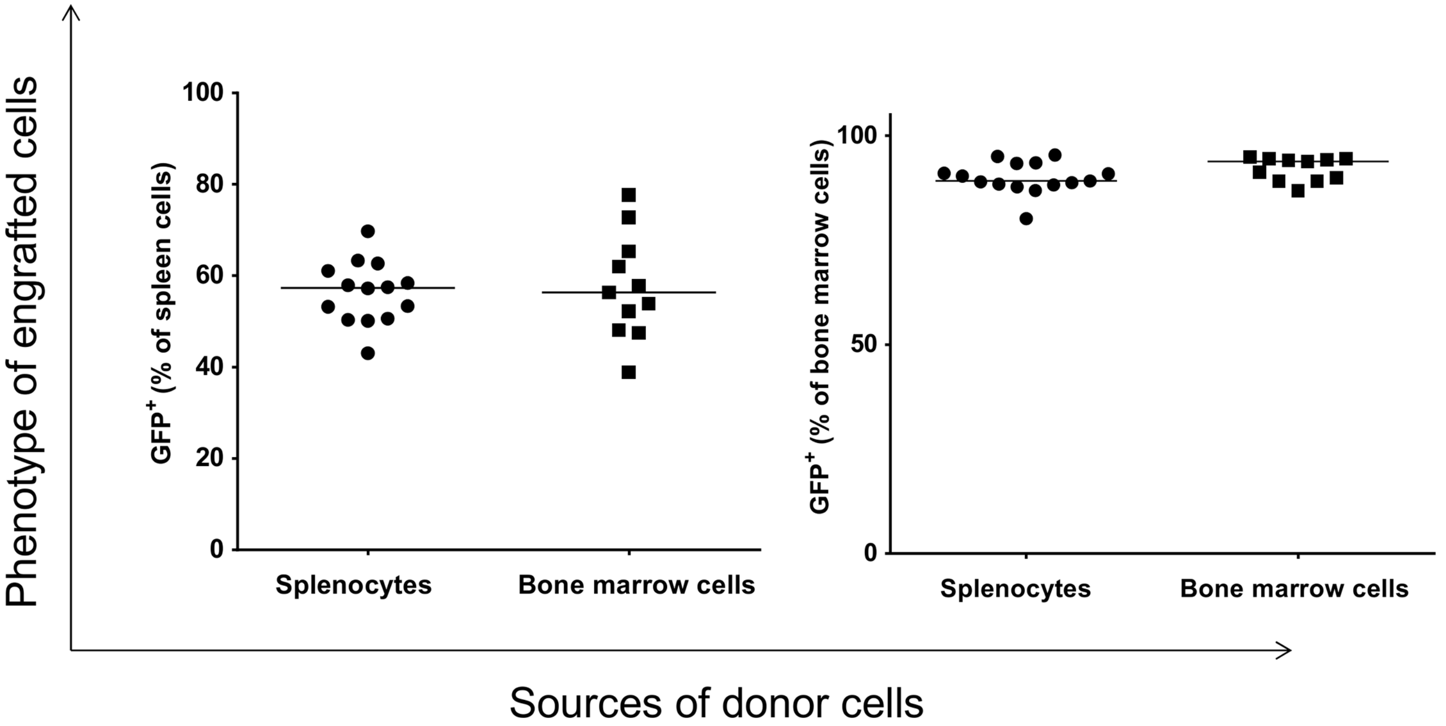

Fig 18. Flow cytometry shows analysis of GFP+ cells in the recipient apo E-/- mice that were transplanted with donor cells from GFP+ mice. Splenoctye and bone marrow cell populations were examined. Apo E-/- recipient mice were transplanted with spleen cells or bone marrow cells from GFP+ donor mice followed by feeding the mice an atherogenic diet for 16 weeks. The mice were euthanized; their spleen cells and bone marrow cells were harvested and analyzed by flow cytometry. The gating strategy to analyze the cell linages was similar to Fig 1. Each data point represents one mouse.

doi:10.1371/journal.pone.0125961.g018

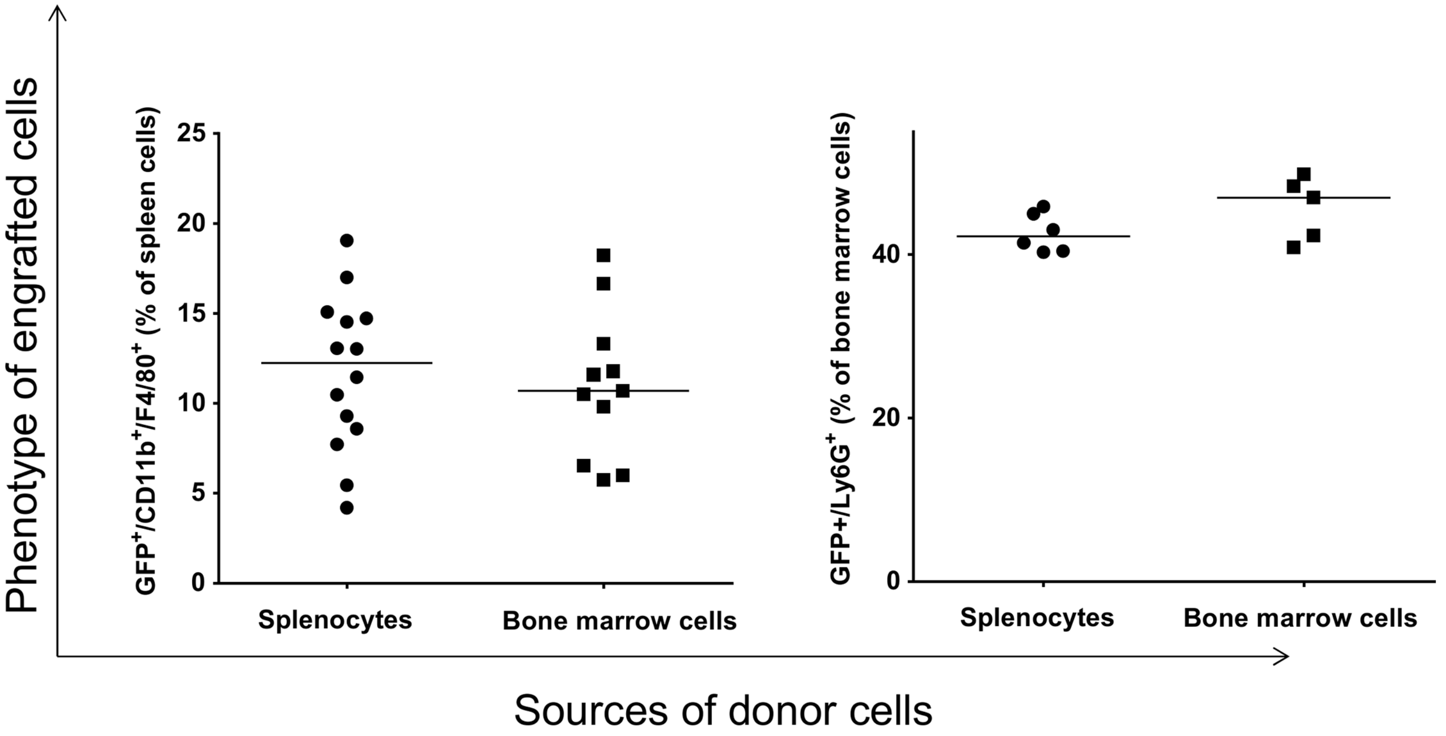

Fig 19. Shows the linage analysis of GFP+ cells in the spleen and bone marrow of recipient mice for their ability to differentiate into monocytes/ macrophages.

doi:10.1371/journal.pone.0125961.g019

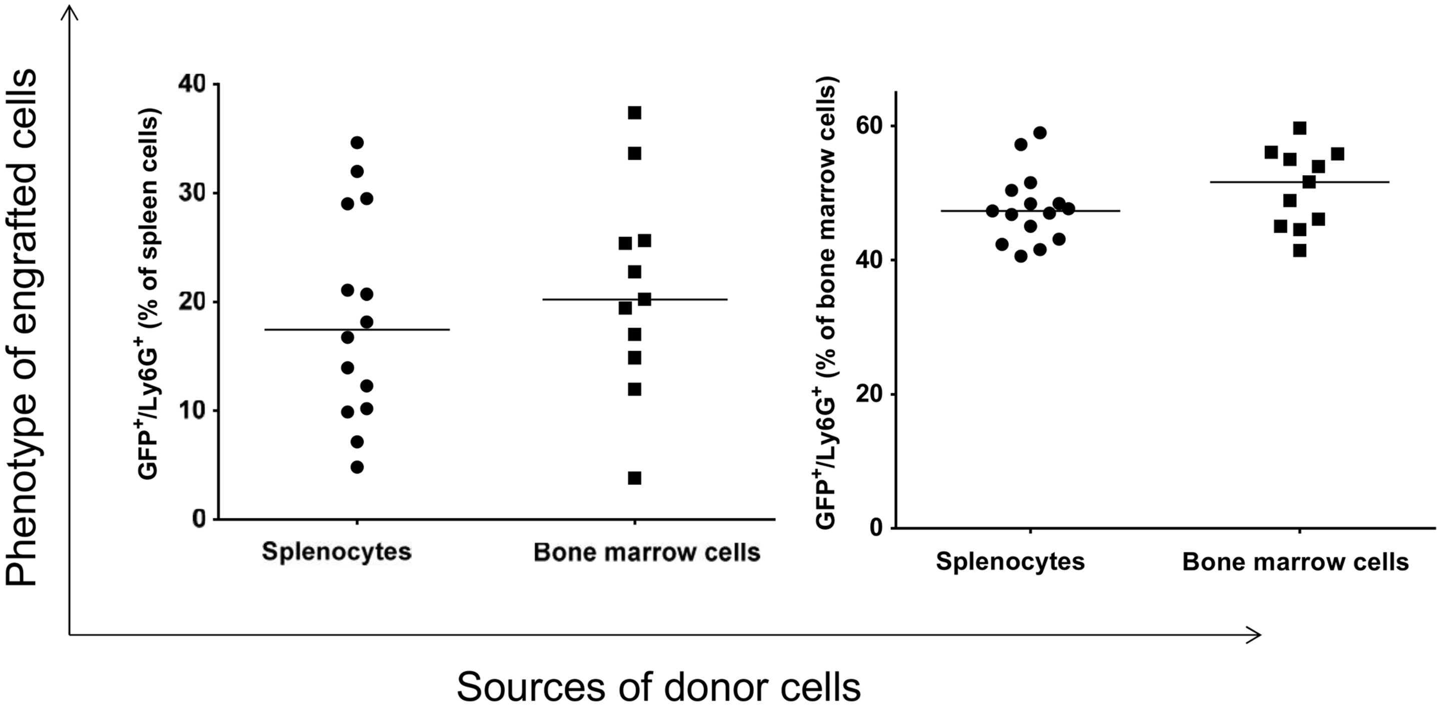

Fig 20. Shows the linage analysis of GFP+ cells in the spleen and bone marrow of recipient mice for their ability to differentiate into neutrophils.

doi:10.1371/journal.pone.0125961.g020

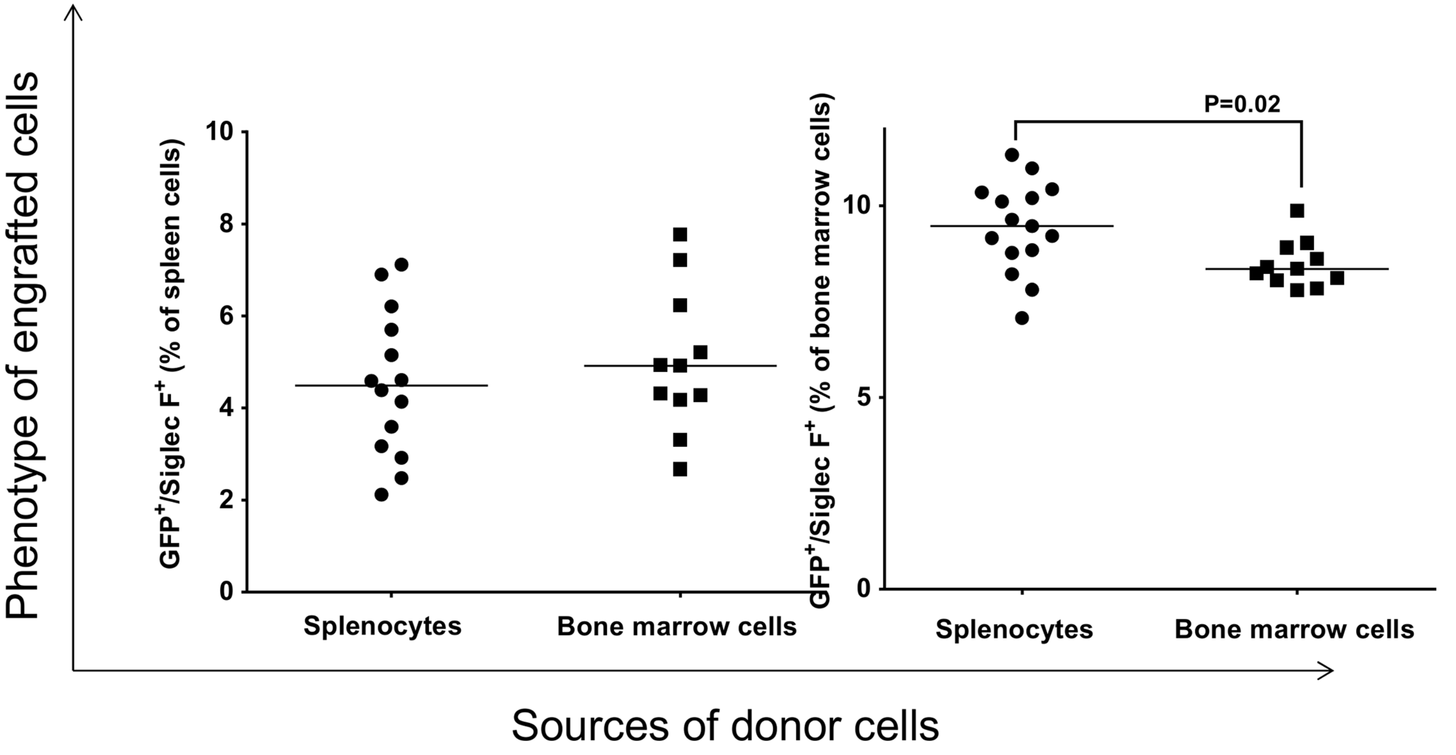

Fig 21. Shows the linage analysis of GFP+ cells in the spleen and bone marrow of recipient mice for their ability to differentiate into eosinophils.

doi:10.1371/journal.pone.0125961.g021

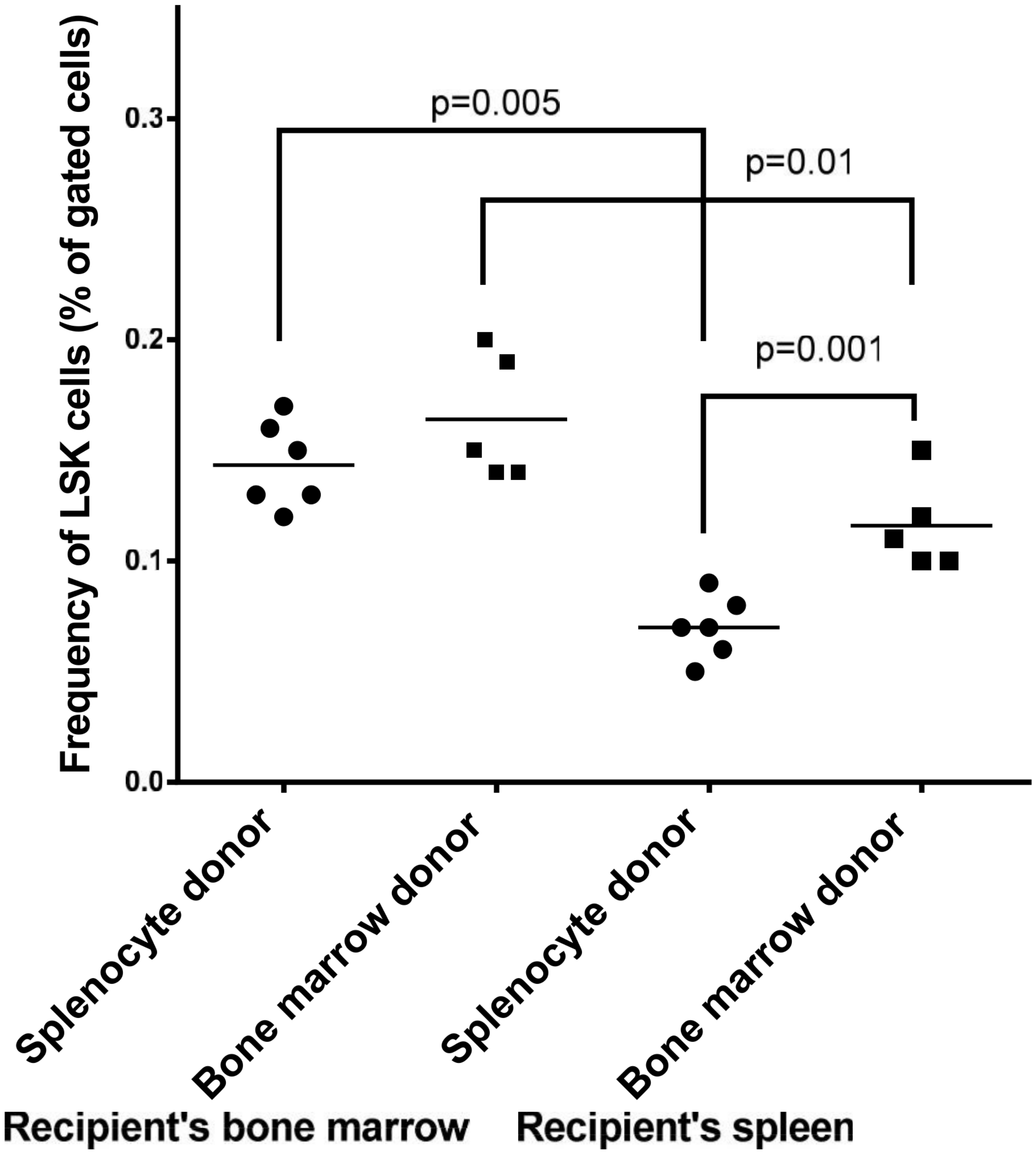

Fig 22. Bone marrow and spleen cells were harvested from apo E-/- mice before feeding the mice an atherogenic diet for 16 weeks. The harvested cells were analyzed for the level of hematopoietic stem (LSK) cells. See Method/Results sections. Each data point represents one mouse.

doi:10.1371/journal.pone.0125961.g022

the hematopoiesis in bone marrow or if it is a "one-way street". To gain a better understanding, we asked whether splenocytes per se are capable of reconstituting bone marrow following the myeloablation. Using GFP^+ donor mice, we observed that donor splenocytes reconstituted bone marrow of lethally-irradiated recipients and the reconstituted mice look healthy and visibly indistinguishable from recipient mice that were transplanted with bone marrow cells. The level of engraftment of donor splenocytes into the bone marrow and spleen of recipient mice was similar to those of donor bone marrow cells.

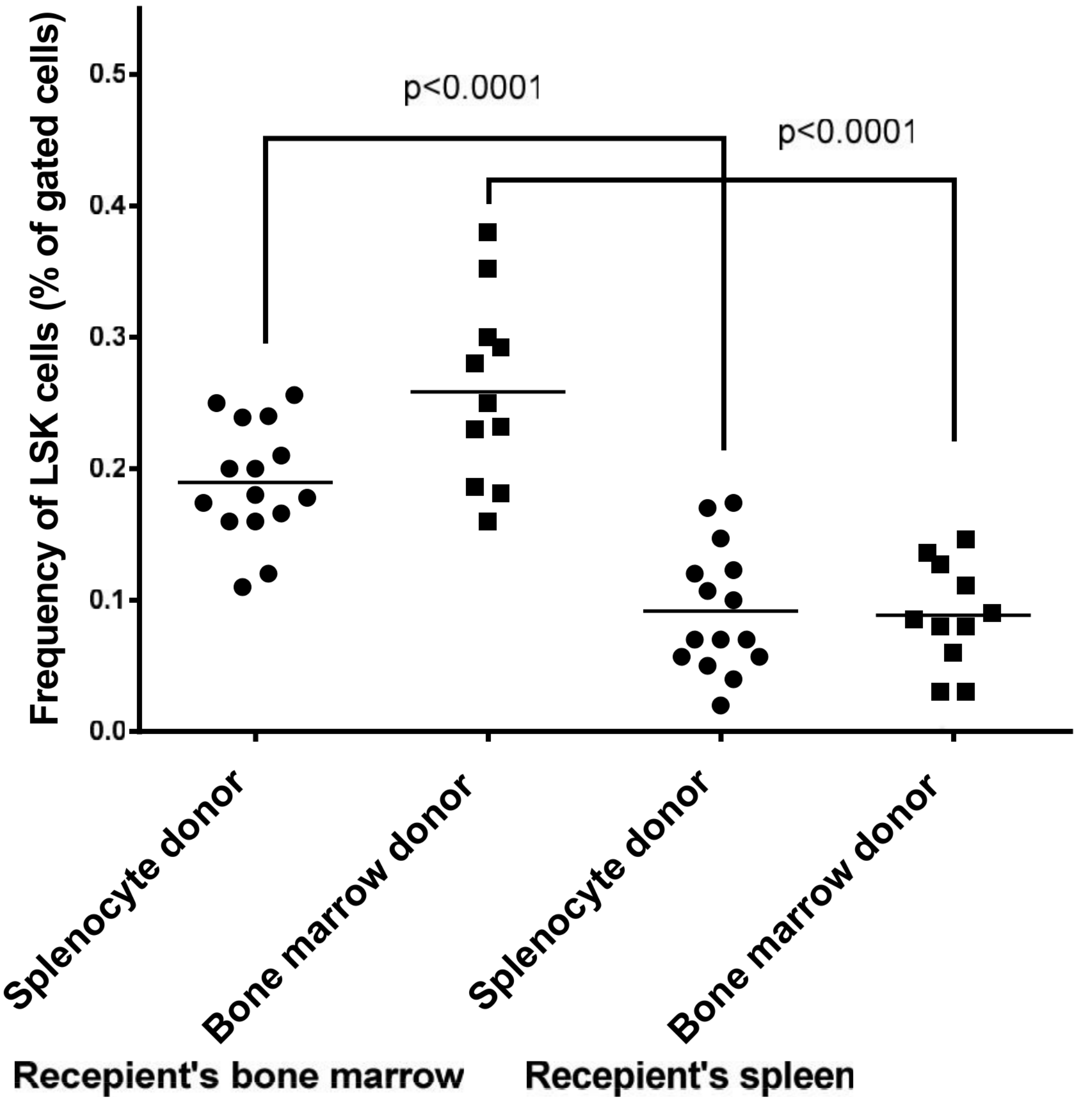

Fig 23. Bone marrow and spleen cells were harvested from apo E-/- mice after feeding the mice an atherogenic diet for 16 weeks. The harvested cells were analyzed for the level of hematopoietic stem (LSK) cells. See Method/Results sections. Each data point represents one mouse.

doi:10.1371/journal.pone.0125961.g023

Although the transplanted spleen cells gave rise to different lineages of myeloid cells in the recipient mice, the level of monocytes/macrophages, eosinophils, and neutrophils in the bone marrow and spleen of recipient was found to be dependent on the tissue origin of donor cells. For example, the frequency of macrophages in the spleens of recipient mice was significantly higher when the donor cells were originated from bone marrow than from spleen. Conversely, significantly more macrophages were found in the bone marrow of recipient mice when the cells are originated from spleen rather than being bone marrow-derived. This indicates that these differences cannot be explained by the level of hematopoietic stem cells in the donor cells. Similarly, the differences do not seem to be related to the irradiation of recipient mice because we used whole body irradiation which uniformly affects both the bone marrow and spleen of recipient mice. These results suggest that the ability of donor cells to differentiate into myeloid lineages is in part determined by the origin of donor cells and it is independent of the

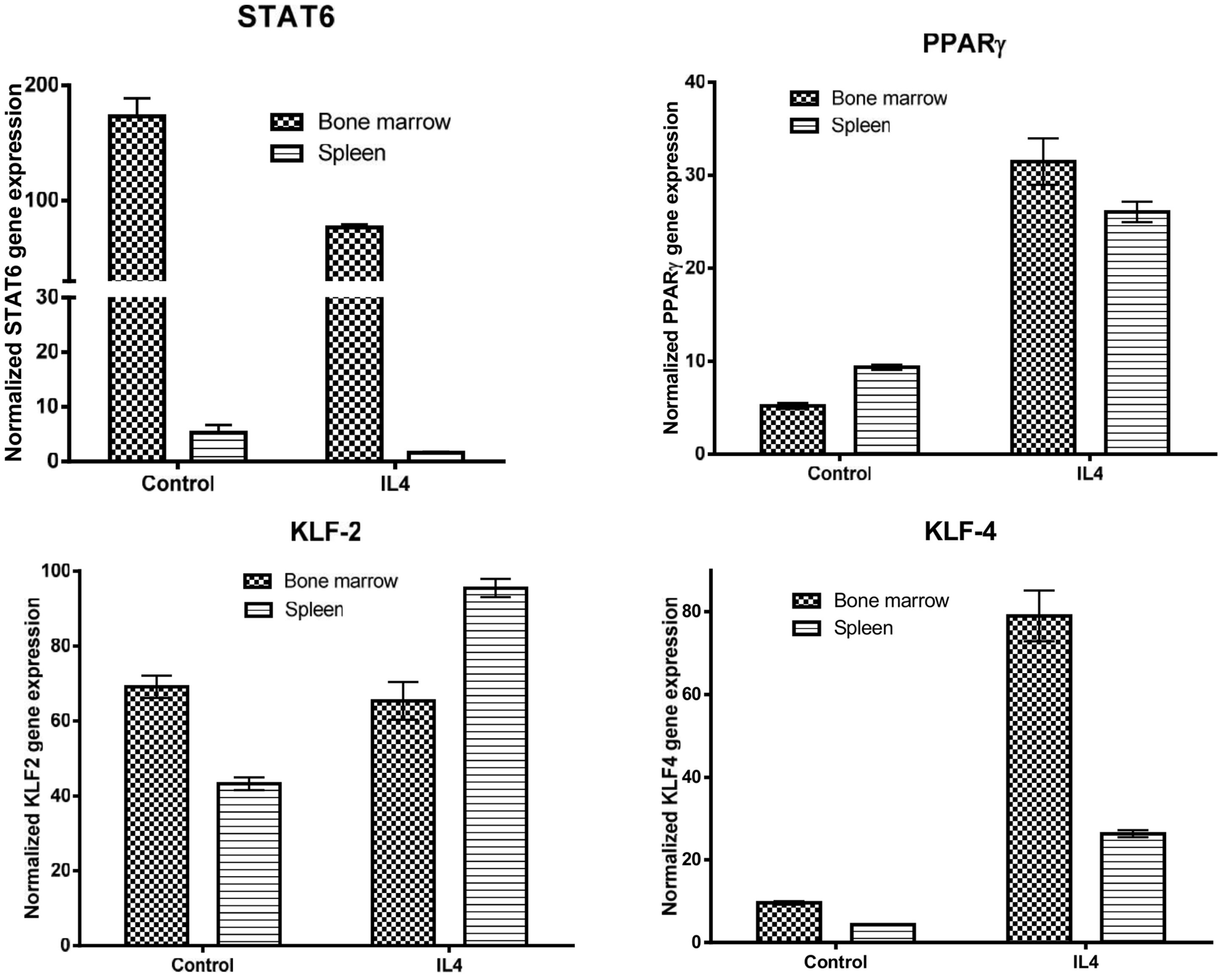

Fig 24. Shows the phenotypic analysis of cultured spleen- and bone marrow-derived macrophages. Splenocytes and bone marrow cells were isolated from GFP$^+$ mice. The cells were seed into culture dishes with splenocytes seeding density 6 times higher than bone marrow cells. The cells were cultured in RPMI/10%serum containing M-CSF. After reaching confluencey, cells were placed for 24 hrs. in the media free of serum or M-CSF. The cells were treated with indicated reagents followed by RNA extraction and analysis. This figure shows the expression of transcription factors known to affect macrophage polarization in the two macrophage sources.

doi:10.1371/journal.pone.0125961.g024

level of stem cells. In line with these observations, we noted that the macrophages isolated from bone marrow exhibit a phenotype that is distinct from those derived from the spleen. These phenotypic differences seem to be stable attribute of cells because they retained their distinct responses to the M1 or M2 agonists when the cells are cultured.

We transplanted an equal number of donor splenocytes and bone marrow cells into the recipient mouse; therefore, the recipients of splenocytes received six times fewer hematopoietic stem cells than the recipients of bone marrow cells. Yet, the recipient of splenocytes were as active as recipient of bone marrow and we could not distinguish any physical differences between the two recipient groups. In line with this observation, we did not detect any differences in the

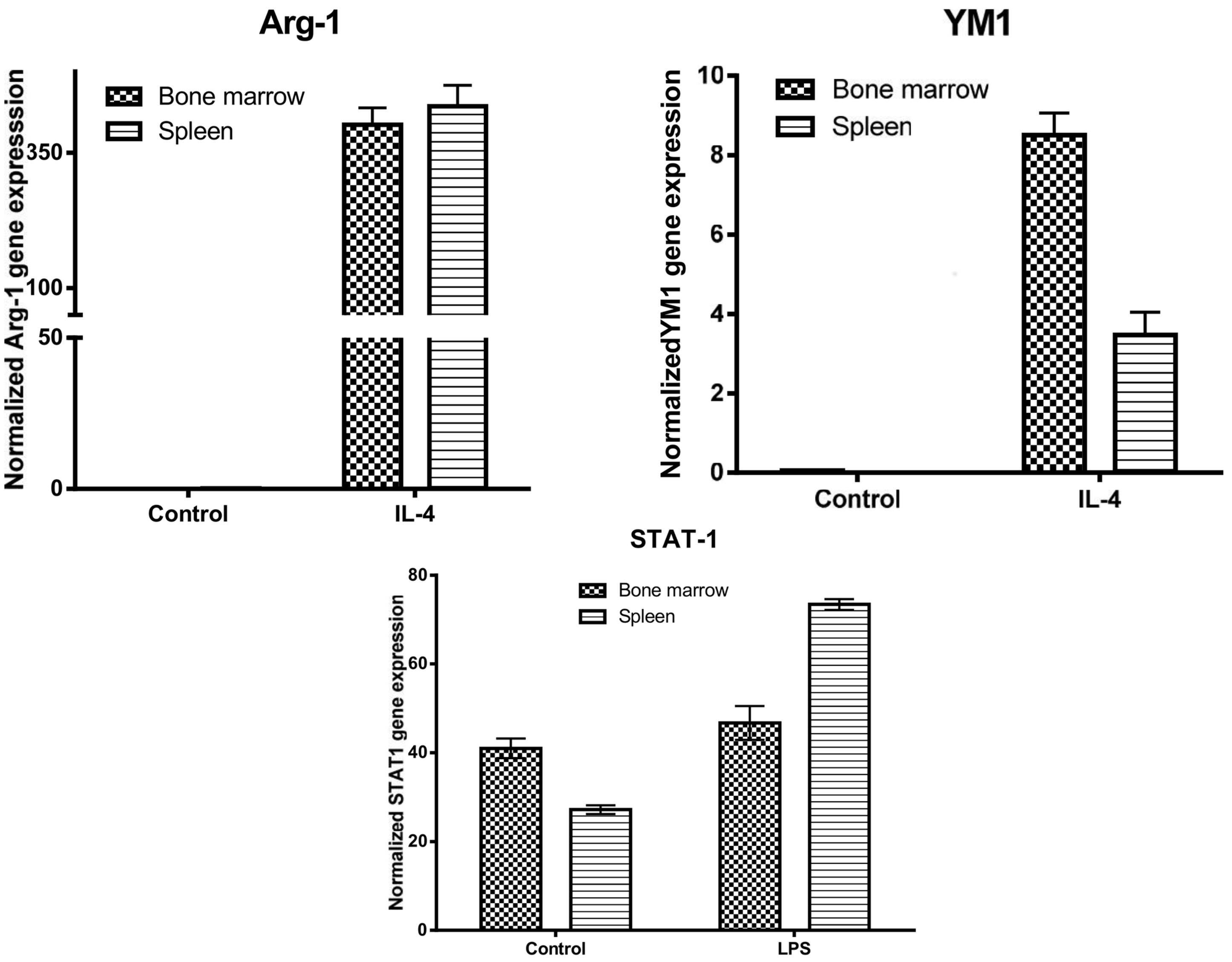

Fig 25. Shows the expression of M2 markers by bone marrow-derived macrophages and spleen-derived macrophages.

doi:10.1371/journal.pone.0125961.g025

atherogenic or atheroprotective activities between the splenocyte and bone marrow cell recipients. Since the end point in the atherosclerosis studies is identical between the two recipient groups and the level of regenerated myeloid cells in the two recipient groups are similar, it is possible that the stem cells derived from spleen may have activity distinct from those from bone marrow. Past studies have shown that the splenic stem cells express Hox11, a well-known embryonic protein [47,48]. Hox11 is a highly conserved homeobox gene that is not found in adult bone marrow, kidney, liver, and salivary gland [4,10]. While during fetal development Hox11 plays a role in the development of these tissues, it is expressed exclusively in adult spleen [47,48]. This reservoir of stem cells is also found in the spleen of adult human [49]. Deletion of Hox11 in mice results in asplenia [47]. The expression of Hox11 in adult splenic cells suggest that these cells are endowed with the ability to descend back to embryonic life in a manner that is different from other adult cells that do not express Hox11 such as bone marrow cells.

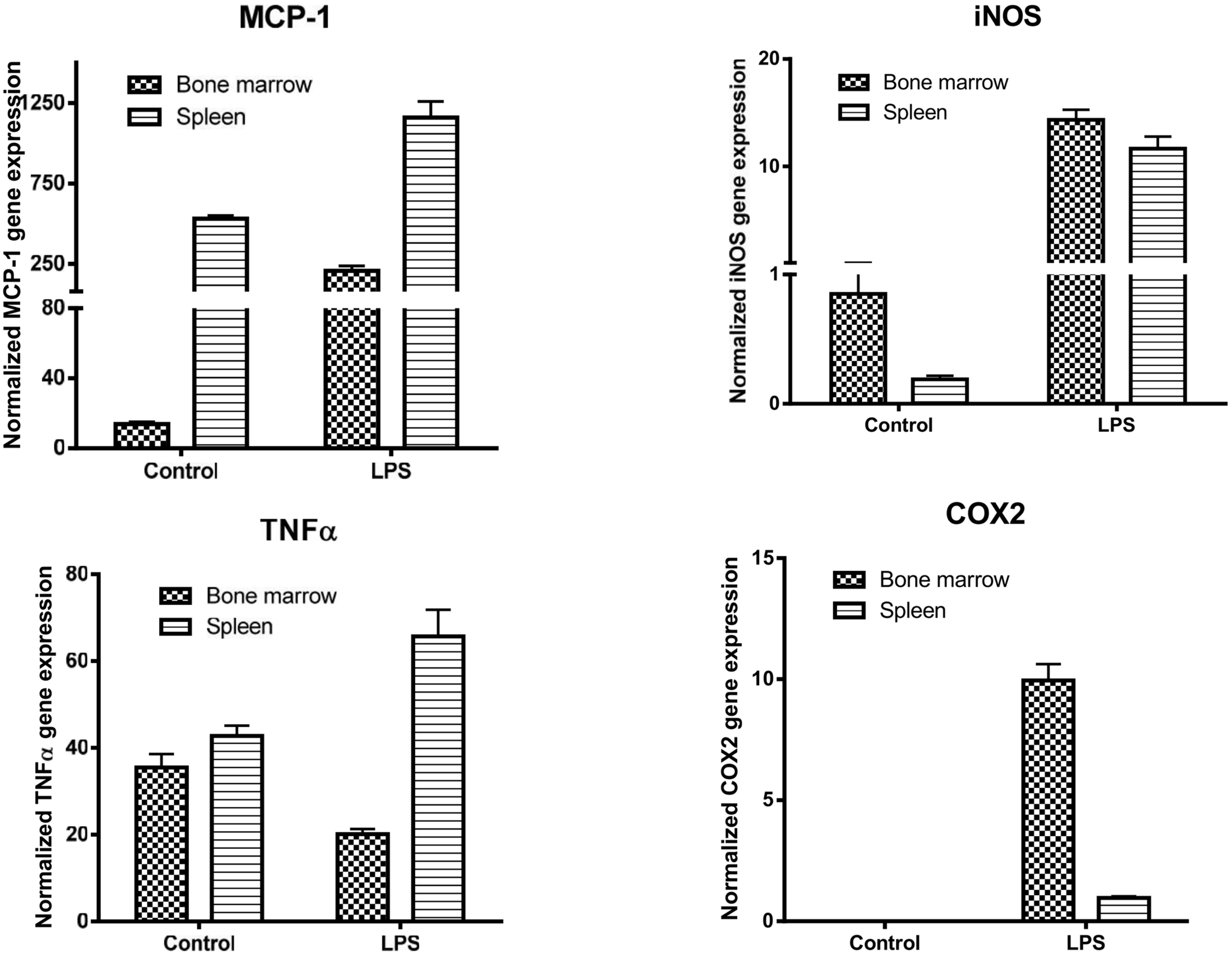

Fig 26. Shows the expression of M1 markers by bone marrow-derived macrophages and spleen-derived macrophages.

doi:10.1371/journal.pone.0125961.g026

While the level of regenerated myeloid cells in the bone marrow and spleen of recipient wild type mice was found to be dependent on the tissue origin of donor cells, this was not the case in apo E-/- recipient mice. We found no significant differences in the level of regenerated myeloid cells in the spleen and bone marrow of apo E-/- recipient mice fed on an atherogenic diet for 16 weeks. Atherosclerosis studies showed that the level of engraftment of donor splenocytes into bone marrow and spleen of apo E-/- recipient mice was similar to the bone marrow donor cells. In addition, the donor splenocytes and bone marrow exhibited a similar atherogenic activity and an atheroprotective effect as determined by the level of lesions between the two recipient groups, the phenotype of the plaques (number of macrophages, smooth muscle cells, and lesion collagen content) as well as the level of cholesterol which is similar between the two donor cell sources. Apo E was found to control proliferation of hematopoietic stem cell, myeloid cell expansion, and monocytosis by interacting with ABCA1/ABCG1 in hematopoietic stem cell, promoting cholesterol efflux and decreasing the cell surface expression and

downstream signaling of the IL-3 receptor [12]. In the transplantation studies, we found no differences in the ability of splenocytes and bone marrow cells to promote and to prevent atherosclerosis despite significantly lower level of hematopoietic stem cells in the spleen than in the bone marrow. This may suggest the hematopoietic stem cells originating from the spleen display a higher level of activity than those derived from the bone marrow.

In the present study, we transplanted bone marrow cells or splenocytes harvested from male mice into lethally-irradiated female mice. This sex-mismatch transplantation allowed us to evaluate cell engraftment by monitoring Y chromosome in the recipient tissues. Past studies have shown that transfer of male cells into female recipients can induce an immune reaction against the Male-Specific Minor Histocompatibility Antigen [50,51]. It has been shown that male cells are rejected 10–12 days after transfer into female recipients ([52]. Therefore, it is possible that the activation of a host-versus-graft rejection immune response may induce an inflammatory response that influences atherogenesis. This, however, is unlikely to alter our conclusion about the effect of transplanted splenocytes in atherosclerosis because we compared atherosclerosis progression/prevention in the splenocytes recipients with those of bone marrow recipients. If the recipients responded to the donor H-Y male antigen, the response would be similar between the two cohorts. In addition, we used lethally-irradiated female mice as recipients, a condition that is fundamentally different from those reported in the literature cited above where the female recipients were normal (i.e. they had not been irradiated). Therefore, we feel that the impact of such sex mismatch transplantation on atherosclerosis is most likely minimal, if any, and it would not affect our conclusion.

In summary, we compared the ability of splenocytes and bone marrow to reconstitute myeloid system of myeloablated mouse. We noted that while the level of engraftment of donor splenocytes into the recipient tissues was similar to donor bone marrow cells, the ability of the spleen-derived cells to differentiate into myeloid cell lineages was different than bone marrow cells. Importantly, we noted that while the level of hematopoietic stem cells in donor spleen cells was markedly lower than the donor bone marrow, the spleen cells displayed similar atherogenic and athero-protective activities as those of bone marrow cells. Cell culture studies showed that phenotype of macrophages derived from spleen is distinct from those of bone marrow. These results suggest that spleen-derived stem cells may be more potent than bone marrow-derived stem cells to reconstitute hematopoietic system of lethally-irradiated mouse.

Supporting Information

S1 Fig. Engraftment of donor cells from male apo E donor mice into female apo E recipient mice as assessed by Y chromosome PCR. DNA was extracted from the indicated recipient mouse tissues, amplified by PCR followed by analysis of PCR products by 1.5% agarose gel. The upper bands (395 bp) correspond to the amplified Y chromosome segment and the lower bands (401 bp) is the internal control (β globin). The male (M) and female (F) brain cDNAs were used as positive and negative controls for Y chromosome. Distilled water was used for PCR control.
(PPTX)

S2 Fig. Phenotype of donor cells in the spleen and bone marrow of male mice. Splenocytes and bone marrow were isolated from male donor mice and stained with the indicated antibodies for flow cytometry analysis. Panel A shows the flow cytometry strategy. Panels B, C, and D show analysis of harvested cells for the level of macrophages, eosinophils, and neutrophils, respectively. These data show that the level of macrophages, eosinophils and neutrophils are significantly higher in the bone marrow than in the spleen of C57BL/6 donor mouse.
(PPTX)

S1 Table. Reagent used in flow cytometry.
(PPTX)

S2 Table. Antibody information.
(PPTX)

S3 Table. PCR primers.
(PPTX)

Acknowledgments

The authors gratefully acknowledge the support of the Heart Foundation, The Eisner Foundation, the Spielberg Foundation, Corday Foundation and the Skirball Foundation. The evolution of this approach was initially supported by program project grant from NHLBI grant R01 HL104068. We are grateful to Patricia Lin for her expertise in flow cytometry.

Author Contributions

Conceived and designed the experiments: BGS PKS. Performed the experiments: LW MY AA LS FL FT MQ AY IW. Analyzed the data: BGS PKS. Wrote the paper: BGS IW PKS.

References

1. Robertson SA, Rowan-Hull AM, Johnson PRV (2008) The spleen—a potential source of new islets for transplantation? Journal of Pediatric Surgery 43: 274–278. doi: 10.1016/j.jpedsurg.2007.10.013 PMID: 18280273
2. Kabelitz D, Geissler EK, Soria B, Schroeder IS, Fändrich F, et al. (2008) Toward cell-based therapy of type I diabetes. Trends in Immunology 29: 68–74. doi: 10.1016/j.it.2007.11.001 PMID: 18182324
3. Faustman DL, Davis M (2010) Stem cells in the spleen: therapeutic potential for Sjogren's syndrome, type I diabetes, and other disorders. Int J Biochem Cell Biol 42: 1576–1579. doi: 10.1016/j.biocel.2010.06.012 PMID: 20601088
4. Kodama S, Davis M, Faustman DL (2005) Diabetes and stem cell researchers turn to the lowly spleen. Sci Aging Knowledge Environ 2005: pe2. PMID: 15659719
5. Tran SD, Kodama S, Lodde BM, Szalayova I, Key S, et al. (2007) Reversal of Sjögren's-like syndrome in non-obese diabetic mice. Annals of the Rheumatic Diseases 66: 812–814. PMID: 17179174
6. Macias MP, Fitzpatrick LA, Brenneise I, McGarry MP, Lee JJ, et al. (2001) Expression of IL-5 alters bone metabolism and induces ossification of the spleen in transgenic mice. The Journal of Clinical Investigation 107: 949–959. PMID: 11306598
7. Lonyai A, Kodama S, Burger D, Faustman DL (2008) Fetal Hox11 expression patterns predict defective target organs: a novel link between developmental biology and autoimmunity. Immunol Cell Biol 86: 301–309. doi: 10.1038/icb.2008.6 PMID: 18301381
8. Lonyai A, Kodama S, Burger D, Davis M, Faustman DL (2008) The promise of Hox11+ stem cells of the spleen for treating autoimmune diseases. Horm Metab Res 40: 137–146. doi: 10.1055/s-2007-1022560 PMID: 18283632
9. Kodama S, Davis M, Faustman DL (2005) The therapeutic potential of tumor necrosis factor for autoimmune disease: a mechanistically based hypothesis. Cell Mol Life Sci 62: 1850–1862. PMID: 15968469
10. Kodama S, Davis M, Faustman DL (2005) Regenerative medicine: a radical reappraisal of the spleen. Trends Mol Med 11: 271–276. PMID: 15949768
11. Bronte V, Pittet Mikael J (2013) The Spleen in Local and Systemic Regulation of Immunity. Immunity 39: 806–818. doi: 10.1016/j.immuni.2013.10.010 PMID: 24238338
12. Murphy AJ, Akhtari M, Tolani S, Pagler T, Bijl N, et al. (2011) ApoE regulates hematopoietic stem cell proliferation, monocytosis, and monocyte accumulation in atherosclerotic lesions in mice. The Journal of Clinical Investigation 121: 4138–4149. doi: 10.1172/JCI57559 PMID: 21968112
13. Robbins CS, Chudnovskiy A, Rauch PJ, Figueiredo J-L, Iwamoto Y, et al. (2012) Extramedullary Hematopoiesis Generates Ly-6Chigh Monocytes That Infiltrate Atherosclerotic Lesions. Circulation 125: 364–374. doi: 10.1161/CIRCULATIONAHA.111.061986 PMID: 22144566

14. Dutta P, Courties G, Wei Y, Leuschner F, Gorbatov R, et al. (2012) Myocardial infarction accelerates atherosclerosis. Nature 487: 325–329. doi: 10.1038/nature11260 PMID: 22763456

15. Leuschner F, Rauch PJ, Ueno T, Gorbatov R, Marinelli B, et al. (2012) Rapid monocyte kinetics in acute myocardial infarction are sustained by extramedullary monocytopoiesis. The Journal of Experimental Medicine 209: 123–137. doi: 10.1084/jem.20111009 PMID: 22213805

16. Ross R (1999) Atherosclerosis—an inflammatory disease. N Engl J Med 340: 115–126. PMID: 9887164

17. Hansson GK (2001) Immune mechanisms in atherosclerosis. Arterioscler Thromb Vasc Biol 21: 1876–1890. PMID: 11742859

18. Jonasson L, Holm J, Skalli O, Bondjers G, Hansson GK (1986) Regional accumulations of T cells, macrophages, and smooth muscle cells in the human atherosclerotic plaque. Arteriosclerosis 6: 131–138. PMID: 2937395

19. Gordon S (2003) Alternative activation of macrophages. Nat Rev Immunol 3: 23–35. PMID: 12511873

20. Gordon S (2007) Macrophage heterogeneity and tissue lipids. The Journal of Clinical Investigation 117: 89–93. PMID: 17200712

21. Mantovani A, Biswas SK, Galdiero MR, Sica A, Locati M (2013) Macrophage plasticity and polarization in tissue repair and remodelling. J Pathol 229: 176–185. doi: 10.1002/path.4133 PMID: 23096265

22. Prieur X (2010) Lipotoxicity in macrophages: evidence from diseases associated with the metabolic syndrome. Biochimica et biophysica acta Molecular and cell biology of lipids 1801: 327–337.

23. Moore KJ (2011) Macrophages in the pathogenesis of atherosclerosis. Cell (Cambridge) 145: 341–355. doi: 10.1016/j.cell.2011.04.005 PMID: 21529710

24. Wang C, Yu X, Cao Q, Wang Y, Zheng G, et al. (2013) Characterization of murine macrophages from bone marrow, spleen and peritoneum. BMC Immunol 14: 6. doi: 10.1186/1471-2172-14-6 PMID: 23384230

25. Wang L, Sharifi BG, Pan T, Song L, Yukht A, et al. (2006) Bone marrow transplantation shows superior atheroprotective effects of gene therapy with apolipoprotein A-I Milano compared with wild-type apolipoprotein A-I in hyperlipidemic mice. J Am Coll Cardiol 48: 1459–1468. PMID: 17010811

26. Malliaras K, Ibrahim A, Tseliou E, Liu W, Sun B, et al. (2014) Stimulation of endogenous cardioblasts by exogenous cell therapy after myocardial infarction. EMBO Molecular Medicine: 760–777. doi: 10.1002/emmm.201303626 PMID: 24797668

27. Li F, Yang M, Wang L, Williamson I, Tian F, et al. (2012) Autofluorescence contributes to false-positive intracellular Foxp3 staining in macrophages: A lesson learned from flow cytometry. Journal of Immunological Methods 386: 101–107. doi: 10.1016/j.jim.2012.08.014 PMID: 23046996

28. Song L, Wang L, Shah PK, Chaux A, Sharifi BG (2010) Bioengineered vascular graft grown in the mouse peritoneal cavity. J Vasc Surg.

29. Sharifi BG, Zeng Z, Wang L, Song L, Chen H, et al. (2006) Pleiotrophin Induces Transdifferentiation of Monocytes Into Functional Endothelial Cells. Arterioscler Thromb Vasc Biol 26: 1273–1280. PMID: 16614316

30. Wang L, Wang W, Shah PK, Song L, Yang M, et al. (2012) Deletion of tenascin-C gene exacerbates atherosclerosis and induces intraplaque hemorrhage in Apo-E-deficient mice. Cardiovascular Pathology.

31. Wang l, Song L, Shah PK, Sharifi BG (2007) Deletion of tenascin gene in apo E mice promotes destabilization of atherosclerotic plaques: The potential role of TN in accumulation of mast cells and over-expression of eotaxin. Circulation 116: 34.

32. Xu XH, Shah PK, Faure E, Equils O, Thomas L, et al. (2001) Toll-like receptor-4 is expressed by macrophages in murine and human lipid-rich atherosclerotic plaques and upregulated by oxidized LDL. Circulation 104: 3103–3108. PMID: 11748108

33. Englen MD, Valdez YE, Lehnert NM, Lehnert BE (1995) Granulocyte/macrophage colony-stimulating factor is expressed and secreted in cultures of murine L929 cells. Journal of Immunological Methods 184: 281–283. PMID: 7658030

34. Moore Kathryn J, Tabas I (2011) Macrophages in the Pathogenesis of Atherosclerosis. Cell 145: 341–355. doi: 10.1016/j.cell.2011.04.005 PMID: 21529710

35. Soehnlein O (2012) Multiple Roles for Neutrophils in Atherosclerosis. Circulation Research 110: 875–888. doi: 10.1161/CIRCRESAHA.111.257535 PMID: 22427325

36. Asai K, Kuzuya M, Naito M, Funaki C, Kuzuya F (1988) Effects of Splenectomy on Serum Lipids and Experimental Atherosclerosis. Angiology 39: 497–504. PMID: 3377269

37. Linton M, Atkinson J, Fazio S (1995) Prevention of atherosclerosis in apolipoprotein E-deficient mice by bone marrow transplantation. Science 267: 1034–1037. PMID: 7863332

38. Boisvert WA, Spangenberg J, Curtiss LK (1995) Treatment of severe hypercholesterolemia in apolipoprotein E-deficient mice by bone marrow transplantation. The Journal of Clinical Investigation 96: 1118–1124. PMID: 7635947

39. Herijgers N, Van Eck M, Groot PHE, Hoogerbrugge PM, Van Berkel TJC (1997) Effect of Bone Marrow Transplantation on Lipoprotein Metabolism and Atherosclerosis in LDL Receptor–Knockout Mice. Arteriosclerosis, Thrombosis, and Vascular Biology 17: 1995–2003. PMID: 9351364

40. Van Eck M, Herijgers N, Yates J, Pearce NJ, Hoogerbrugge PM, et al. (1997) Bone Marrow Transplantation in Apolipoprotein E–Deficient Mice: Effect of ApoE Gene Dosage on Serum Lipid Concentrations, (β)VLDL Catabolism, and Atherosclerosis. Arteriosclerosis, Thrombosis, and Vascular Biology 17: 3117–3126. PMID: 9409301

41. Gordon S, Taylor PR (2005) Monocyte and macrophage heterogeneity. Nat Rev Immunol 5: 953–964. PMID: 16322748

42. Gordon S, Martinez FO (2010) Alternative activation of macrophages: mechanism and functions. Immunity 32: 593–604. doi: 10.1016/j.immuni.2010.05.007 PMID: 20510870

43. Liu G, Xia X, Gong S, Zhao Y (2006) The macrophage heterogeneity: difference between mouse peritoneal exudate and splenic F4/80+ macrophages. J Cell Physiol 209: 341–352. PMID: 16883572

44. Ricardo SD, van Goor H, Eddy AA (2008) Macrophage diversity in renal injury and repair. The Journal of Clinical Investigation 118: 3522–3530. doi: 10.1172/JCI36150 PMID: 18982158

45. Johns JL, Christopher MM (2012) Extramedullary Hematopoiesis: A New Look at the Underlying Stem Cell Niche, Theories of Development, and Occurrence in Animals. Veterinary Pathology Online 49: 508–523. doi: 10.1177/0300985811432344 PMID: 22262354

46. Kim CH (2010) Homeostatic and pathogenic extramedullary hematopoiesis. J Blood Med 1: 13–19. doi: 10.2147/JBM.S7224 PMID: 22282679

47. Dear TN, Colledge WH, Carlton MB, Lavenir I, Larson T, et al. (1995) The Hox11 gene is essential for cell survival during spleen development. Development 121: 2909–2915. PMID: 7555717

48. Kanzler B, Dear TN (2001) Hox11 Acts Cell Autonomously in Spleen Development and Its Absence Results in Altered Cell Fate of Mesenchymal Spleen Precursors. Developmental Biology 234: 231–243. PMID: 11356032

49. Dieguez-Acuna FJ, Gygi SP, Davis M, Faustman DL (2007) Splenectomy: a new treatment option for ALL tumors expressing Hox-11 and a means to test the stem cell hypothesis of cancer in humans. Leukemia 21: 2192–2194. PMID: 17713543

50. Goulmy E, Termijtelen A, Bradley BA, Van Rood JJ (1976) ALLOIMMUNITY TO HUMAN H-Y. The Lancet 308: 1206.

51. Greenfield A, Scott D, Pennisi D, Ehrmann I, Ellis P, et al. (1996) An H-YDb epitope is encoded by a novel mouse Y chromosome gene. Nat Genet 14: 474–478. PMID: 8944031

52. Tyznik AJ, Bevan MJ (2007) The Surprising Kinetics of the T Cell Response to Live Antigenic Cells. Journal of immunology (Baltimore, Md: 1950) 179: 4988–4995.
